# Supplementary material for: Strong electron-phonon coupling driven pseudogap modulation and density-wave fluctuations in a correlated polar metal
Source: Nat Commun. 2023 Sep 18;14:5769. doi: 10.1038/s41467-023-41460-x (PMC10507017; doi:10.1038/s41467-023-41460-x)
Supplement: Supplementary file 1 — Supplementary Information [file 41467_2023_41460_MOESM1_ESM.pdf]

Strong Electron-Phonon Coupling driven Pseudogap Modulation and Density-Wave Fluctuations in a Correlated Polar Metal

Huaiyu (Hugo) Wang[α,*,1,7], Yihuang Xiong[*,1,2], Hari Padma[1], Yi Wang[1], Ziqi Wang[1], Romain Claes[3], Guillaume Brunin[4], Lujin Min[1], Rui Zu[1], Maxwell T. Wetherington[1], Yu Wang[5,6], Zhiqiang Mao[5,6], Geoffroy Hautier[2,3], Long-Qing Chen[1], Ismaila Dabo[β,1], Venkatraman Gopalan[γ,1]
[1]Materials Research Institute and Department of Material Science & Engineering, Pennsylvania State University, University Park, Pennsylvania, 16802, USA
[2]Thayer School of Engineering, Dartmouth College, 14 Engineering Drive, Hanover, New Hampshire, 03755, USA
[3]Institute of Condensed Matter and Nanosciences (IMCN), Université catholique de Louvain, Chemin des Étoiles 8, B-1348, Louvain-la-Neuve, Belgium
[4]Matgenix, Gozée, Belgium
[5]2D Crystal Consortium, Material Research Institute, Pennsylvania State University, University Park, Pennsylvania, 16802, USA
[6]Department of Physics, Pennsylvania State University, University Park, Pennsylvania 16802, USA
[7]Present address: Stanford Institute for Materials and Energy Sciences, SLAC National Accelerator Laboratory, Menlo Park, California, 94025, USA

[*]These authors contributed equally to this work.
[α]hxw230@psu.edu
[β]ixd4@psu.edu
[γ]vgopalan@psu.edu

Supplementary Note 1: Summary of the previous incorrect symmetry assignment of phonons in $Ca_3Ru_2O_7$
Supplementary Note 2: Raman spectra collected with different laser lines.
Supplementary Note 3: Phonon assignments and the agreement between the Density Functional Theory (DFT) and the Raman data
Supplementary Note 4: Description of the fitting procedure of the B_2 phonons and its robustness with respect to the background subtraction
Supplementary Note 5: Evidence to support five phonon modes in B_2 spectra between 350 to 450 cm^{-1} through processing raw spectra and chemical doping.
Supplementary Note 6: Sensitivity tests of functionals and their impact on phonon energy
Supplementary Note 7: Fitting results of the temperature dependent Raman spectra
Supplementary Note 8: Joint Density of States results from the DFT calculations
Supplementary Note 9: Detailed analysis of B_2^P and B_2^M phonons
Supplementary Note 10: Raman Vertex
Supplementary Note 11: Hund's Rule description
Supplementary Note 12: Electronic Raman Background related analysis
Supplementary Note 13: Optical Selection Rule on d-d transition between neighboring Ru sites
Supplementary Note 14: Lack of static charge density wave phase in $Ca_3Ru_2O_7$ from structural evidence
Supplementary Note 15: Discussion on density wave fluctuation lifetime
Supplementary Note 16: Calculation of laser heating
Supplementary Note 17: Electronic band structure and phonon eigen mode calculated from DFT

Supplementary Table 1. 2mm character table.
Supplementary Table 2. Raman phonon mode assignment.
Supplementary Table 3. Summarized maximum errors in fitting of B_2 Raman spectra considering all background subtraction choices.
Supplementary Table 4. The summary of B_2 phonon mode energy in cm^{-1} for pure and 3% Ti doped $Ca_3Ru_2O_7$.
Supplementary Table 5. The 1 Q ($\text{Å}\sqrt{\text{amu}}$) eigen vector of B_2^P phonon with initial atomic position of moving atoms respectively.

Supplementary Table 6. The 1 Q (Å$\sqrt{\mathrm{amu}}$) eigen vector of B_2^M phonon with initial atomic position of moving atoms respectively.

Supplementary Figure 1. Wavelength dependent Raman spectra.

Supplementary Figure 2. Low temperature Raman spectra.

Supplementary Figure 3. Raman spectra fitting as a function of background subtraction.

Supplementary Figure 4. Finding peaks from processing Raman raw data.

Supplementary Figure 5. Tuning the peak frequency by doping Ti atoms.

Supplementary Figure 6. Phonon assignment in pure and Ti doped $Ca_3Ru_2O_7$.

Supplementary Figure 7. Fitting results of 10K $Ca_3Ru_2O_7$ B_2 Raman spectra with different models.

Supplementary Figure 8. Sensitivity tests of functionals (PBEsol, LDA, and PBE) and their impacts on potential energy surface ΔU vs. mean-square displacement Q.

Supplementary Figure 9. Temperature dependent fitting results of $B_2^{(1)}$ to $B_2^{(9)}$.

Supplementary Figure 10. Temperature dependent fitting results of $B_2^{(10)}$ to $B_2^{(15)}$.

Supplementary Figure 11. Temperature dependent fitting results of $A_1^{(1)}$ to $A_1^{(9)}$.

Supplementary Figure 12. Temperature dependent fitting results of $A_1^{(10)}$ to $A_1^{(18)}$.

Supplementary Figure 13. Joint density of states calculation results.

Supplementary Figure 14. Illustration of atom labels.

Supplementary Figure 15. d ratio and in-plane bond angles of octahedra 1 and 2 modulated by B_2^{M} and B_2^{P} phonons.

Supplementary Figure 16. In-plane hopping integral modulated by B_2^{P} and B_2^{M} phonon.

Supplementary Figure 17. Temperature dependence of B_2^{P} phonon amplitude and pseudogap dip area.

Supplementary Figure 18. Temperature dependence of $B_2^{(4)}$ and $B_2^{(6)}$ and their correlation with charge transfer hump area.

Supplementary Figure 19. Temperature-dependence of bond length and bond angles in $Ca_3Ru_2O_7$.

Supplementary Figure 20. Volume perturbation by B_2^{P} phonon mode.

Supplementary Figure 21. Temperature-dependence of density wave fluctuation lifetime compared with phonon energy of B_2^{P} phonon.
Supplementary Figure 22. DFT calculation of electronic band structures of AFM-*b* and AFM-*a* phases.
Supplementary Figure 23. Eigen modes from VASP frozen phonon calculation.

Supplementary Note 1: Summary of the previous incorrect symmetry assignment of phonons in $Ca_3Ru_2O_7$

The symmetry of $Ca_3Ru_2O_7$ in the previous work is treated as $A2_1ma$ with lattice parameter $a<b<c$[1]. However, the correct symmetry assignment is $Bb2_1m$ with lattice parameter $a<b<c$[2]. This means the 2-fold axis was erroneously assigned to the shorter in-plane a-axis in Ref. 1. If we rewrite the **Table 1** *m2m* character table to *2mm* character table according to Ref. 1, we will get the wrong table shown in **Supplementary Table 1**. This would result in the wrong assignment of $\bar{z}(xy)z$ as the B_1 phonon modes. This means the usage of point groups in Ref. 1, even based on wrong crystal structure, is still wrong. In particular, the statement in Ref. 1 that "$A2_1ma$ structure (or $Cmc2_1$ with a being the long axis)" is misleading. The correct switching from $A2_1ma$ to $Cmc2_1$ is that (a,b,c) switches to (c,a,b). So a in $Cmc2_1$ corresponds to b in $A2_1ma$, which according to the wrong crystal structure is the medium axis. In Ref. 1, the broad feature in $\bar{z}(xy)$z was interpreted as a single phonon with Fano line shape which softens from 435 cm^{-1} to 413 cm^{-1}. The analysis and mode assignment in the Ref. 1 is one of the many studies[3-6] that misinterpreted this broad asymmetric feature in $\bar{z}(xy)z$ between 400 cm^{-1} and 450 cm^{-1}. Since the physical interpretation in previous works are based on wrong phonon assignment, their conclusions based on this assignment are not well-supported.

Supplementary Table 1. 2mm character table. The wrong character table of 2*mm* symmetry for $Ca_3Ru_2O_7$ Raman assignment following the assignment of the 2-fold rotation symmetry element in Ref. 1, with the Raman polarizability symmetry of $\bar{z}(xx)z$ and $\bar{z}(xy)z$ corresponding to A_1 and B_1 following this wrong table.

2mm	1	2	m(xy)	m(xz)		
A_1	1	1	1	1	x	x^2, y^2,z^2
A_2	1	1	-1	-1	R_x	yz
B_1	1	-1	1	-1	y, R_z	xy
B_2	1	-1	-1	1	z, R_y	xz

Supplementary Note 2: Raman spectra collected with different laser lines.

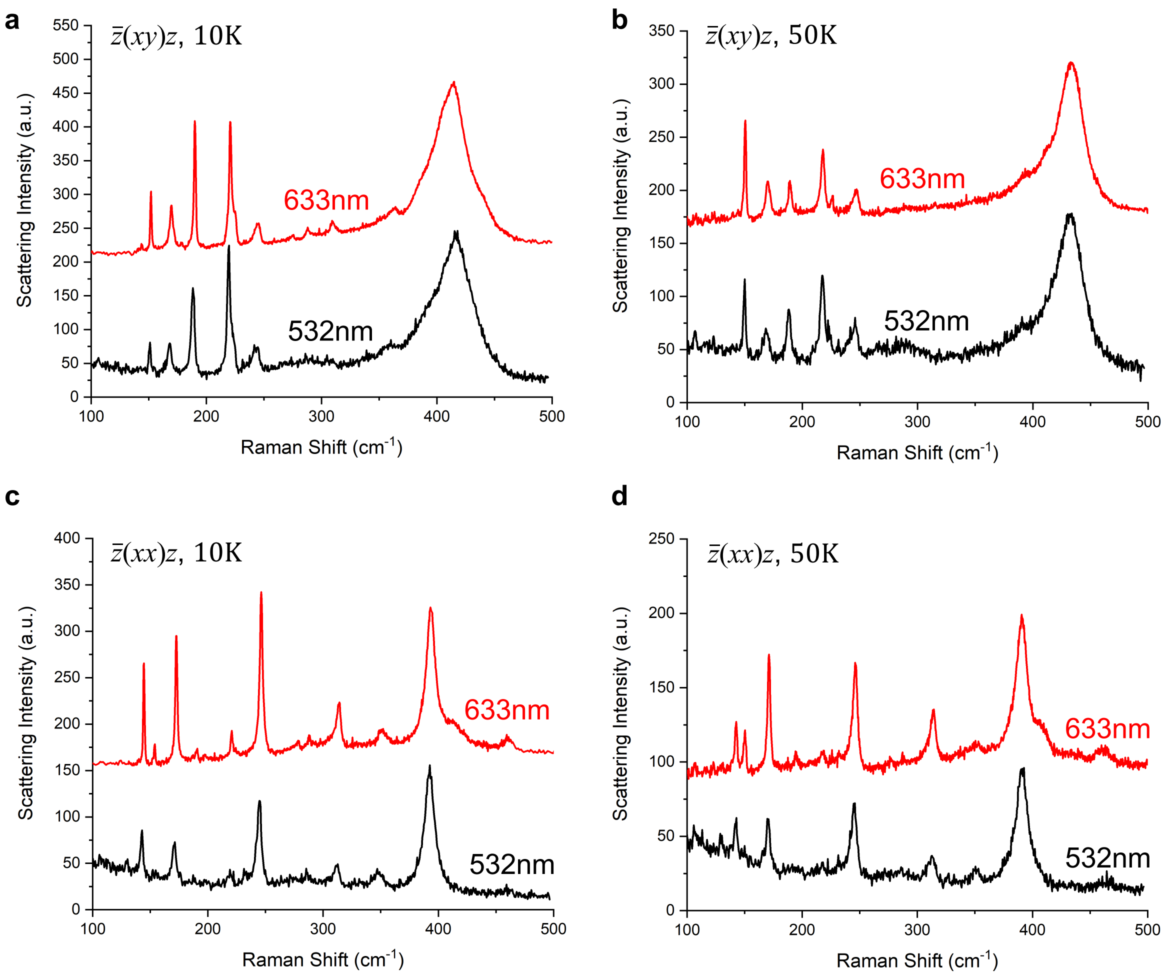

Supplementary Figure 1. Wavelength dependent Raman spectra. The excitation wavelength dependence of Raman spectra with 532nm and 633nm laser light and 1800 gr/cm grating in the $\bar{z}(xy)z$ geometry at (a) 10K in the AFM-*b* phase, and at (b) 50K in the AFM-*a* phase, as well as in the $\bar{z}(xx)z$ geometry at (c) 10K in the AFM-*b* phase, and at (d) 50K in the AFM-*a* phase. The 633nm $\bar{z}(xx)z$ and $\bar{z}(xy)z$ spectra were collected using 600 μW power for 1 hour (12 reps of 300s) and 2 hours (24 reps of 600s) respectively, and 532nm $\bar{z}(xx)z$ and $\bar{z}(xy)z$ spectrum were collected using 400 μW power for 20 mins (10 reps of 120s) and 45 mins (9 reps of 300s) respectively.

The wavelength dependence of the peaks between 350 and 500 cm^{-1} in the XY spectra is minimal.

Supplementary Note 3: Phonon assignments and the agreement between the Density Functional Theory (DFT) and the Raman data

Supplementary Table 2. Raman phonon mode assignment. First principles DFT calculation of the phonon energies compared with the experimental observations.

Mode	AFM-*b* DFT	AFM-*b* Exp.	AFM-*a* DFT	AFM-*a* Exp.	Mode	AFM-*b* DFT	AFM-*b* Exp.	AFM-*a* DFT	AFM-*a* Exp.
$A_1^{(1)}$	145	145	144	143	$B_2^{(1)}$	140	152	140	150
$A_1^{(2)}$	148	154	147	150	$B_2^{(2)}$	170	170	170	170
$A_1^{(3)}$	164	173	163	171	$B_2^{(3)}$	173	179	172	
$A_1^{(4)}$	193	198	193	195	$B_2^{(4)}$	207	190	207	189
$A_1^{(5)}$	211		212		$B_2^{(5)}$	220	221	221	218
$A_1^{(6)}$	230	221	228	217	$B_2^{(6)}$	232	225	230	219
$A_1^{(7)}$	244	246	245	246	$B_2^{(7)}$	262	244	263	246
$A_1^{(8)}$	264	276	266	276	$B_2^{(8)}$	270	275	270	274
$A_1^{(9)}$	273	291	273	291	$B_2^{(9)}$	299	288	297	290
$A_1^{(10)}$	301	313	301	314	$B_2^{(10)}$	305	310	303	315
$A_1^{(11)}$	327		326		$B_2^{(11)}$	362	361	355	354
$A_1^{(12)}$	345	351	344	349	$B_2^{(12)}$	378	388	378	393
$A_1^{(13)}$	387	393	382	391	$B_2^{(13)}$	386	403	383	412
$A_1^{(14)}$	407	414	401	410	$B_2^{(14)}$	400	415	402	427
$A_1^{(15)}$	445	460	442	460	$B_2^{(15)}$	409	440	405	435
$A_1^{(16)}$	550		545		$B_2^{(16)}$	511		493	
$A_1^{(17)}$	600	581	595		$B_2^{(17)}$	569		558	
$A_1^{(18)}$	614	628	615	624	$B_2^{(18)}$	584		582	

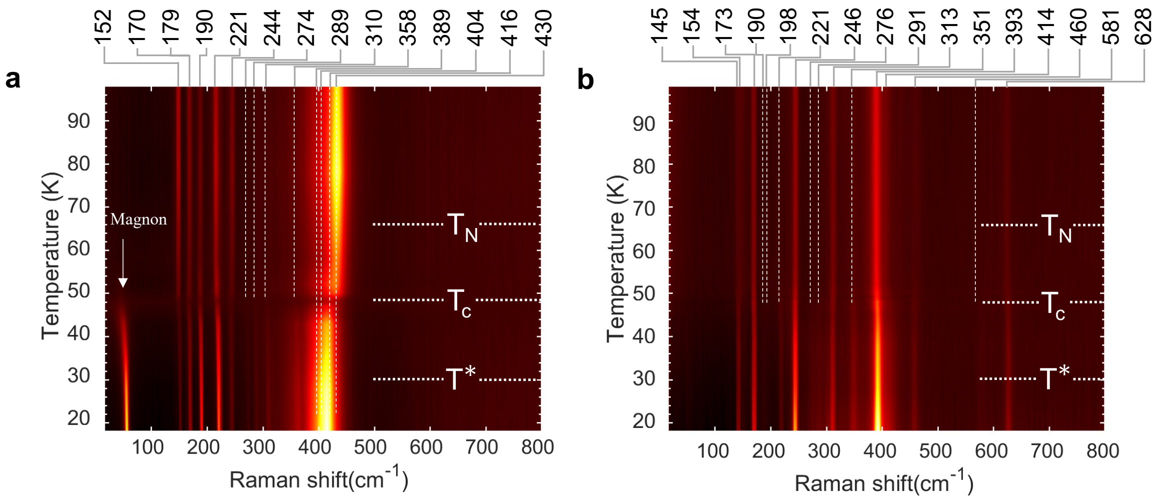

Supplementary Figure 2. Low temperature Raman spectra. Temperature dependent Raman spectra for (a) the B_2 modes in $\bar{z}(xy)z$ and (b) the A_1 modes in $\bar{z}(xx)z$.

Supplementary Note 4: Description of the fitting procedure of the B_2 phonons and its robustness with respect to the background subtraction and model choice

A broad asymmetric feature between 350 and 500 cm^{-1} in the $\bar{z}(xy)z$ Raman spectrum exists in the explored temperature range. From the DFT prediction, there are five B_2 phonon modes that reside in this energy range. For the interpretation of the asymmetric peaks, a common practice is to use the Fano line shape. However, using the five Fano line shapes to fit the Raman spectrum in this window causes overfitting. In this work, we make use of the Savitzky-Golay finite impulse response smoothing filter[10] to extrapolate the background of the Raman spectra to account for the electronic continuum contribution to the background. Before applying the filter, we first make use of a moving average filter with a binning window of 3 cm^{-1} to smooth out the the noise. There are two parameters to control the Savitzky-Golay filter, the polynomial order n, and frame length, L. We fix the polynomial order to be n=1 and change the frame length to control the background shape. Furthermore, the extracted background is filtered to be smaller than the raw signal. This process is reiterated until convergence.

We tested the above background subtraction filter on the 633nm 1800gt/mm Raman data, and the results are shown in **Supplementary Fig. 3**. The L was taken from 7 to 19 and only odd numbers are accepted. The smaller the L, the more background that is subtracted as the electron continuum contribution. When the length is taken as 19, the background subtraction is almost linear between 350 and 500 cm^{-1}. In all wavenumber ranges, we can fit the data with reasonable error bars given the same initial conditions. The initial condition is chosen in such a way that is guided by the DFT calculation. The peak position fitting results are summarized in **Supplementary Table 3**, and the error bar is calculated by taking the global minimum and maximum value of the parameters. Overall, a large variation in the background range does not affect the position fitting of the phonon

modes (except for the $B_2^{(11)}$ mode at 50K), meaning the fitting results are reasonably robust against background choices.

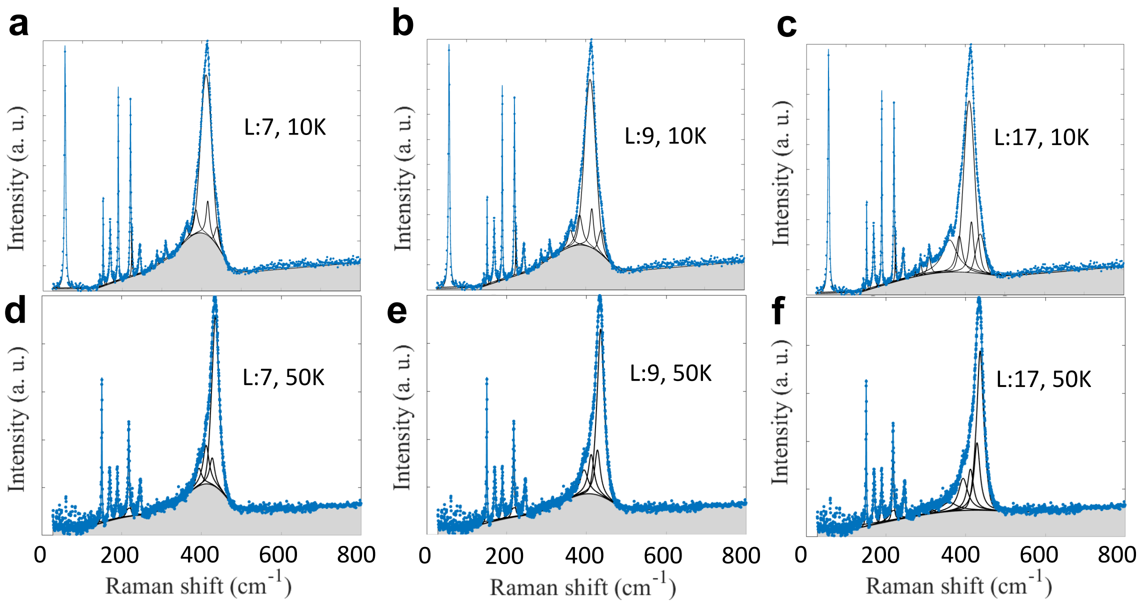

Supplementary Figure 3. Raman spectra fitting as a function of background subtraction. The background subtraction results (grey shaded area) together with total fitting (blue lines) and individual peaks fitting (black lines) of the $\bar{z}(xy)z$ raw data (blue dots). The temperature of each dataset is at (a-c) 10K and (d-f) 50K with different L parameters.

Supplementary Table 3. Summarized maximum errors in fitting of B_2 Raman spectra considering all background subtraction choices. The summary results for the B_2 phonon energy in cm^{-1} as well as the error bars calculated by accounting for all the background choices considered, and then compared with the DFT results.

Mode	AFM-*b* DFT	AFM-*b* Exp	AFM-*a* DFT	AFM-*a* Exp
$B_2^{(1)}$	140	151.92±0.03	138	150.40±0.03
$B_2^{(2)}$	170	169.65±0.09	165	170.13±0.14
$B_2^{(3)}$	173	178.60±0.55	171	
$B_2^{(4)}$	207	190.02±0.02	204	189.25±0.14
$B_2^{(5)}$	220	220.79±0.03	220	217.71±0.08
$B_2^{(6)}$	232	224.72±0.14	231	218.77±2.48
$B_2^{(7)}$	262	244.28±0.19	262	246.34±0.25
$B_2^{(8)}$	270	274.64±0.83	269	274.13±1.71
$B_2^{(9)}$	299	288.14±0.45	294	290.01±2.46
$B_2^{(10)}$	305	309.62±0.37	302	315.17±0.84
$B_2^{(11)}$	362	360.77±1.64	355	353.71±16.42
$B_2^{(12)}$	376	387.97±1.29	379	393.46±2.02
$B_2^{(13)}$	386	402.57±1.93	382	411.50±1.04
$B_2^{(14)}$	400	414.95±0.80	401	427.03±2.10
$B_2^{(15)}$	409	440.47±2.06	404	435.03±1.16
$B_2^{(16)}$	511		493	
$B_2^{(17)}$	559		558	
$B_2^{(18)}$	584		583	

Supplementary Note 5: Evidence to support five phonon modes in *B_2* spectra between 350 to 450 cm^{-1} from processing raw spectra and chemical doping.

The analysis below is free of peak fitting and should provide confidence in our claim of five peaks between 350 and 500 cm^{-1}. The first and second derivatives of a function can sensitively reveal the presence of a peak as illustrated by a simple Lorentzian in **Supplementary Fig. 4a-c**. For a single peak, the first derivative should first increase and then decrease, and the point where it crosses zero is the peak position. If peaks are congested, the first derivative might not cross zero and the second derivative of the peak (**Supplementary Fig. 4c**) can help identify the peak by showing local minimum dips at the peak position. The $Ca_3Ru_2O_7$ B_2 Raman profile at 10K (**Supplementary Fig. 4d**) has many peaks between 350 and 500 cm^{-1} and we can make use of the above-mentioned method to find peaks. The first derivative of the Raman profile (**Supplementary Fig. 4e**) reveals several positions. Many of the first derivative value does cross zero, indicating there are peaks in the Raman spectra; however, three of them does not cross zero (~388, 403 and 440 cm^{-1}). The second derivative of the Raman profile (**Supplementary Fig. 4f**) does show a dip at these three Raman shift positions, confirming that they are shoulder peaks and should be considered during fitting. The identified peaks agree well with our fitting functions choice.

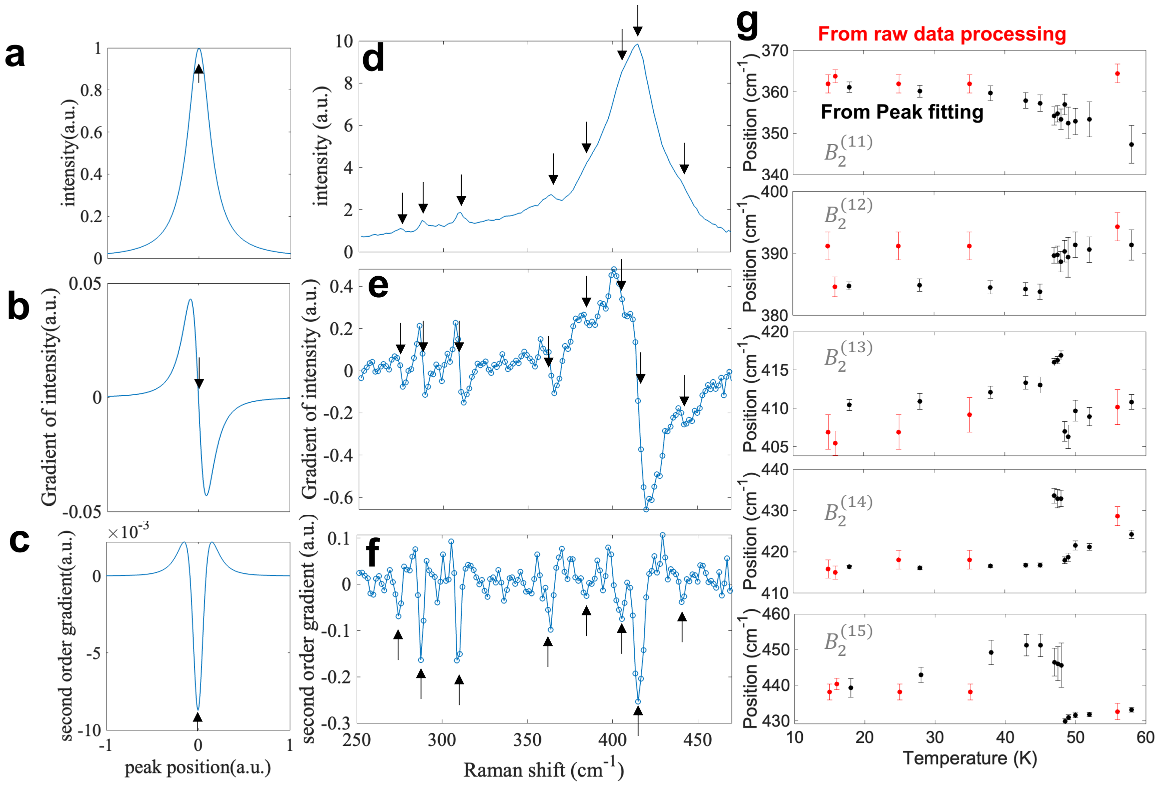

Supplementary Figure 4. Finding peaks from processing Raman raw data. (a) Lorentzian peak profile. (b) First derivative of peak intensity with respect to peak position. (c) Second derivative of peak intensity with respect to peak position. (d) $Ca_3Ru_2O_7$ B_2 Raman profile at 10K. (e) The first derivative of data in (d) with respect to Raman shift. (f) The second derivative of data in (d) with respect to Raman shift. The black arrows indicate the feature of a peak. (g) The peak finding method has been applied to 1800 grating data (red) to obtain the energy of $B_2^{(11)}$ to $B_2^{(15)}$ phonon peaks. The black data points are phonon energy obtained from peak fitting of 600 grating data points.

Furthermore, we use external stimulus to disentangle the peaks by tuning the chemical potential in $Ca_3Ru_2O_7$ via doping 3% Ti to replace some of the Ru sites. The doping of Ti does not involve

a symmetry change, with the space group remaining in $Bb2_1m$ after doping[7]. Since there is no crystallographic point group change before and after Ti doping, the number of phonon modes in B_2 irrep should remain the same between pure vs. the Ti-doped crystal structures. However, the magnetic structure at 10K change from AFM-*b* phase in pure $Ca_3Ru_2O_7$ to G-AFM phase in 3% Ti doped $Ca_3(Ru_{1-x}Ti_x)_2O_7$.[7] The congested profiles between 350 to 500 cm^{-1} in AFM-b phase (**Supplementary Fig. 5b**) is tuned to split into at least four distinct peaks in G-AFM phase (**Supplementary Fig. 5c**), with a possible fifth that may be too weak to clearly distinguish. This provides evidence that further supports our identification of several phonon peaks in this region in pure $Ca_3Ru_2O_7$. Furthermore, we examine the fitting across the measured Raman shift range up to 800 cm^{-1} and we can find one-to-one correspondence between B_2 modes in 3% Ti doped $Ca_3Ru_2O_7$ phases, and those in pure $Ca_3Ru_2O_7$ phases (**Supplementary Fig. 6** and **Supplementary Table 4**). The doping effect on phonon energy is quite interesting and we plan to further study it.

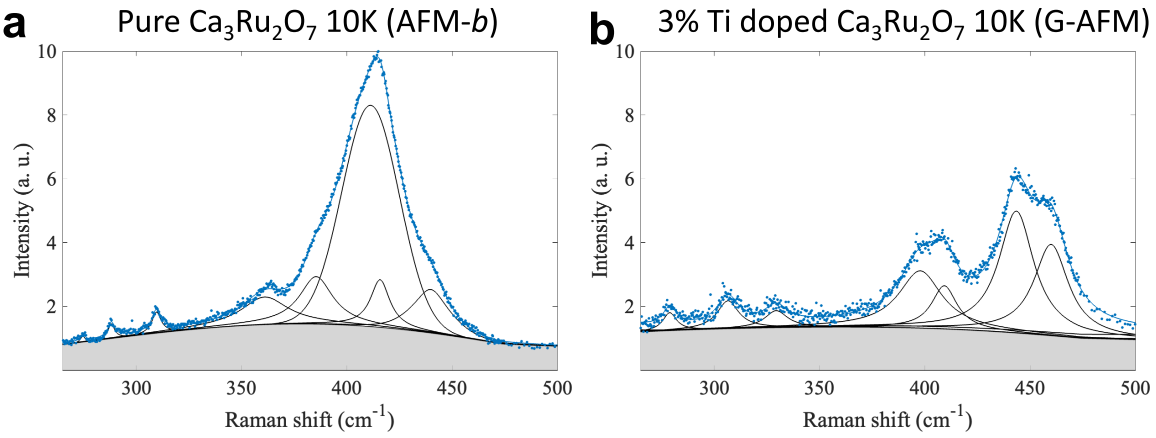

Supplementary Figure 5. Tuning the peak frequency by doping Ti atoms. The fitting results of individual B_2 phonon peaks (black solid line) overlayed with overall fitting results (blue solid

line), background subtraction (grey filled area, L is 11, see **Supplementary Note 4**) and raw data (blue dots) of (a) pure $Ca_3Ru_2O_7$ at 10K, and (b) 3% Ti doped $Ca_3Ru_2O_7$ at 10K.

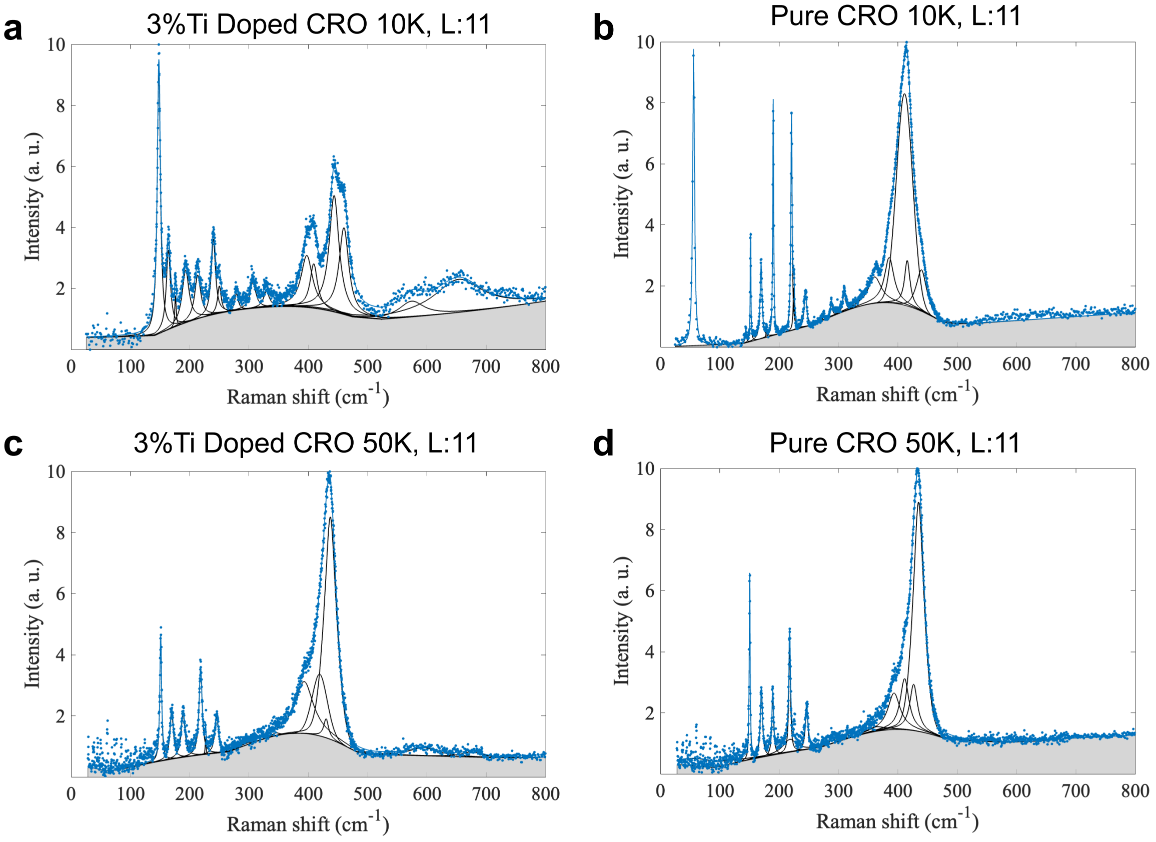

Supplementary Figure 6. Phonon assignment in pure and Ti doped $Ca_3Ru_2O_7$. The fitting results of individual B_2 phonon peaks (black solid line) overlayed with overall fitting results (blue solid line), background subtraction (grey filled area, see **Supplementary Note 4**) and raw data (blue dots) of (a) 3% Ti doped $Ca_3Ru_2O_7$ at 10K, (b) pure $Ca_3Ru_2O_7$ at 10K, (c) 3% Ti doped $Ca_3Ru_2O_7$ at 50K (AFM-a phase), (d) pure $Ca_3Ru_2O_7$ at 50K (AFM-a phase).

Supplementary Table 4. The summary of B_2 phonon mode energy in cm^{-1} for pure and 3% Ti doped $Ca_3Ru_2O_7$.

	3%Ti doped Ca3Ru2O7 G-AFM (10K)	Pure Ca3Ru2O7 AFM-b (10K)	3%Ti doped Ca3Ru2O7 AFM-a (50K)	Pure Ca3Ru2O7 AFM-a (50K)
$B_2^{(1)}$	148	152	151	150
$B_2^{(2)}$	164	170	170	170
$B_2^{(3)}$	176	179		
$B_2^{(4)}$	193	190	189	189
$B_2^{(5)}$	214	221	218	218
$B_2^{(6)}$	240	225	218	219
$B_2^{(7)}$	250	244	226	246
$B_2^{(8)}$	279	275	245	274
$B_2^{(9)}$	307	288	294	290
$B_2^{(10)}$	329	310	317	315
$B_2^{(11)}$		361	351	353
$B_2^{(12)}$	397	388	395	393
$B_2^{(13)}$	409	403	414	412
$B_2^{(14)}$	443	415	430	427
$B_2^{(15)}$	460	440	437	435
$B_2^{(16)}$				
$B_2^{(17)}$	574		583	
$B_2^{(18)}$	654		677	

The robustness of the five peak positions to different background subtraction schemes is discussed in **Supplementary Note 4.** If we assume minimal hybridization between phonons and electronic background, then the fitting result (see **Supplementary Fig. 7a**) is poor at near 360 cm^{-1}. We also must address the non-constant Raman background from electron-phonon coupling by considering a distorted profile (see **Supplementary Fig. 7b** and **Supplementary Note 4**). Since the affected

area is composed of five peaks (see arguments above), using Fano-line shapes on five peaks leads to overfitting. We therefore make use of background subtraction to account for the electron-phonon coupling contribution in the Raman background. We also demonstrate the fitting result using a single Fano line shape to describe Raman profile between 380-450 cm^{-1} following the previous Raman paper[1] (see **Supplementary Fig. 7c**); this fitting quality is rather poor as highlighted by black arrows.

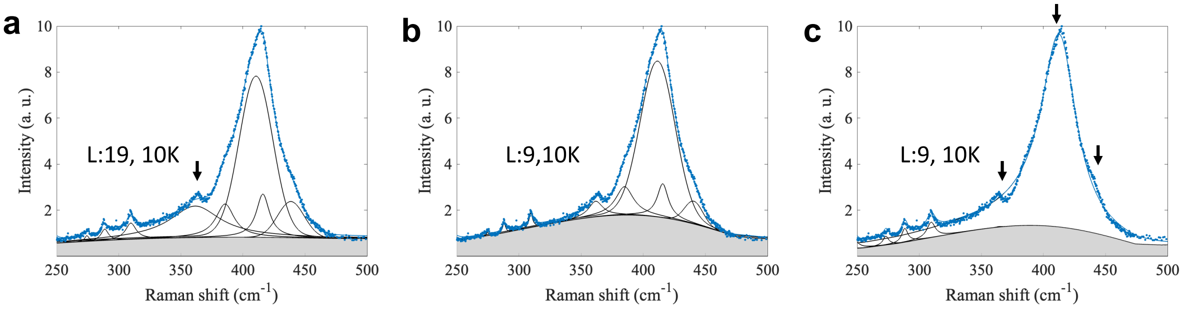

Supplementary Figure 7. Fitting results of 10K $Ca_3Ru_2O_7$ B_2 Raman spectra with different models. The fitting results of individual phonon peaks (black solid line) overlayed with overall fitting results (blue solid line), background subtraction (grey filled area, see **Supplementary Note 4**) and raw data (blue dots) for (a) five phonon peaks between 350 and 500 cm^{-1} with small phonon peak electronic continuum mixture in background and (b) large mixture in background, and (c) single Fano peak between 350 and 500 cm^{-1}. The arrows indicate where the fitting is poor.

Supplementary Note 6: Sensitivity tests of functionals and their impact on phonon energy

To address the potential uncertainties on how different DFT functionals can affect the predicted phonon energy in $Ca_3Ru_2O_7$, we investigated two additional functionals: PBE and PBEsol that is optimized for solid-state properties to compare with our previous LDA data. We computed the potential energy surfaces (PES) by displaying the atoms along the $B_2^{(13)}$ and $B_2^{(15)}$ phonon modes within the harmonic approximations and fitted the PES with quadratic functions. For consistency, on-site Hubbard repulsion of 1.2 eV on Ru 4*d* orbitals and spin-orbit coupling are included throughout. For the ease of comparison, we reference the energy with respect to the ground-state energy of AFM-b. The results are summarized in **Supplementary Figure 8**. For the phonon modes that are of interest in $Ca_3Ru_2O_7$, the PES is relatively insensitive to the choice of functionals as the coefficients of the quadratic terms show small variations across the functionals. More importantly, $B_2^{(15)}$ consistently shows larger coefficients compared to $B_2^{(13)}$, which indicates that the phonon frequency of $B_2^{(15)}$ would be larger than $B_2^{(13)}$ regardless of the choice of functionals. Therefore, it is expected that order of $B_2^{(13)}$ and $B_2^{(15)}$ will remain unaltered and would not impact our phonon mode assignments. We also highlighted that LDA has been shown to perform well for vibrational properties for oxides[8,9].

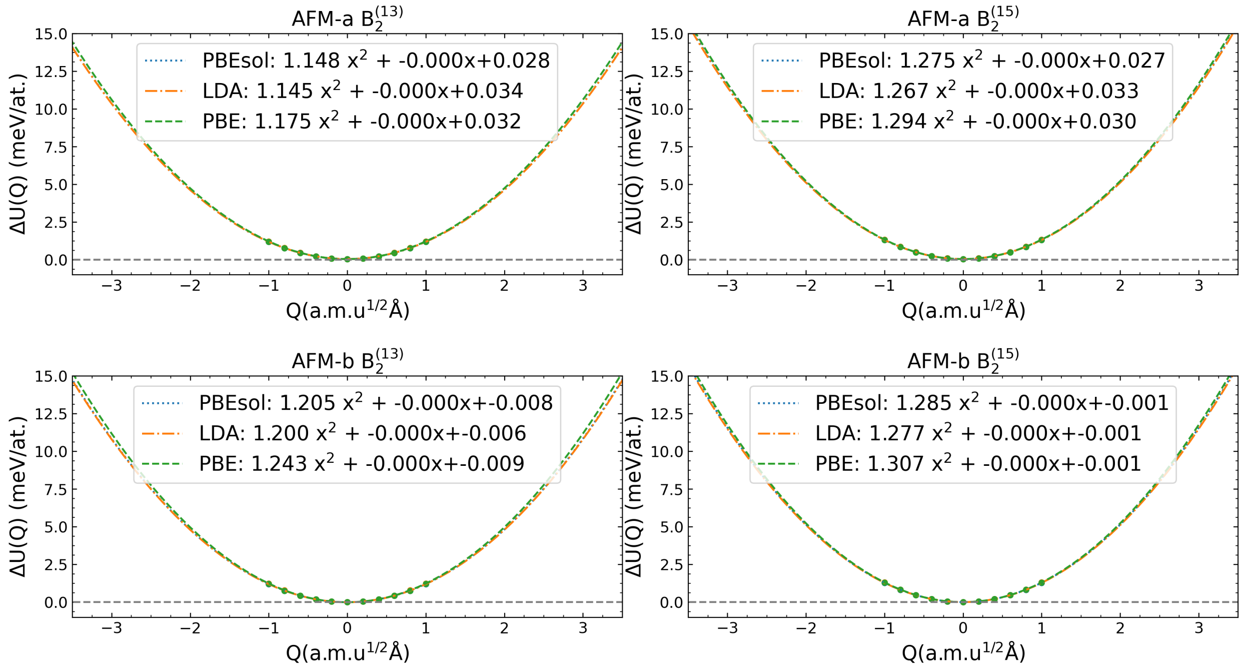

Supplementary Figure 8. Sensitivity tests as a function of the exchange-correlation functionals (PBEsol, LDA, and PBE) and their impact on the potential energy surface Δ*U* with respect to the normal mode coordinate *Q*. (a) AFM-a $B_2^{(13)}$, (b) AFM-a $B_2^{(15)}$, (c) AFM-b $B_2^{(13)}$, and (d) AFM-b $B_2^{(15)}$.

Supplementary Note 7: Fitting results of the temperature dependent Raman spectra

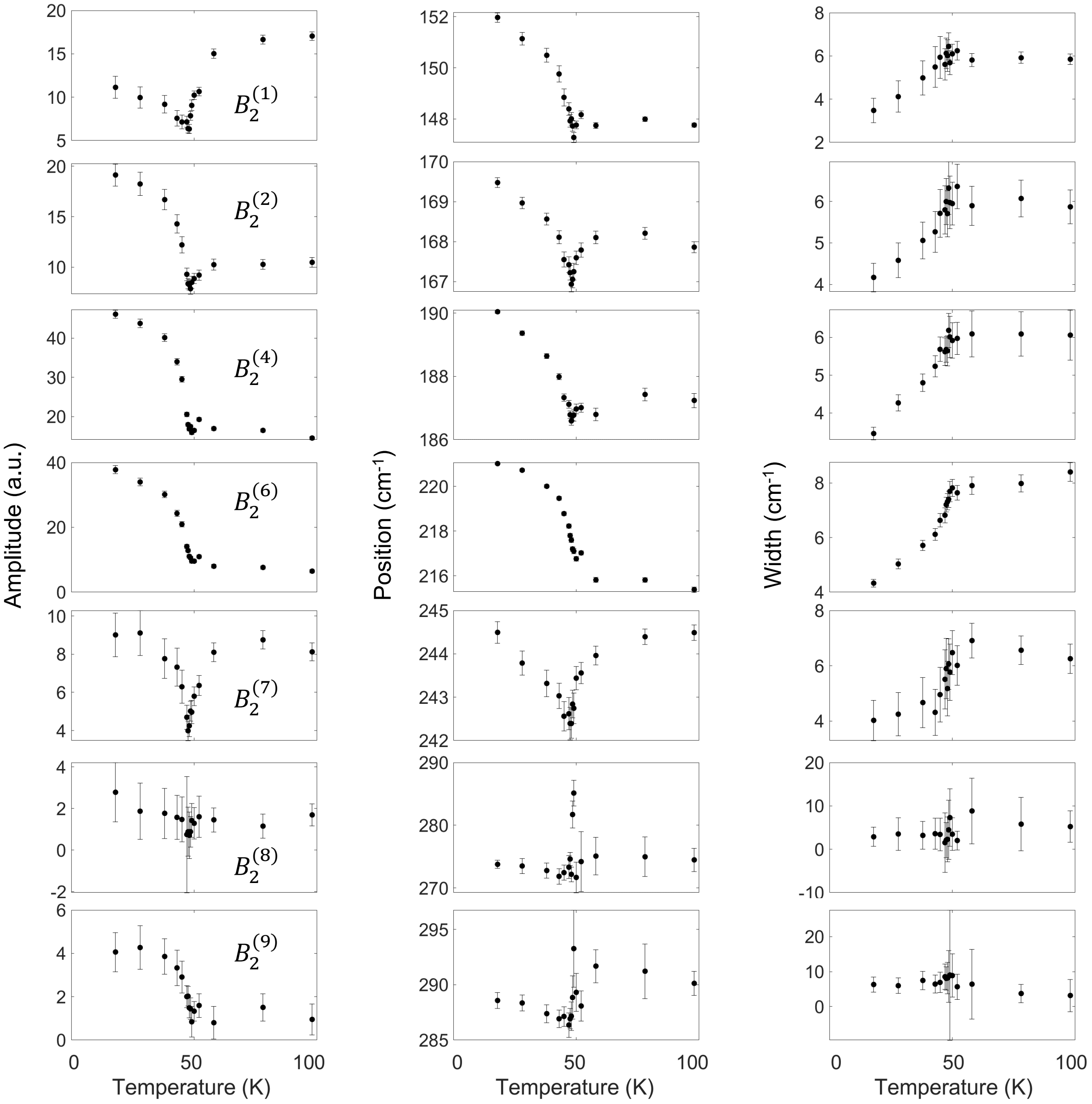

Supplementary Figure 9. Temperature dependent fitting results of $B_2^{(1)}$ to $B_2^{(9)}$. Fitting parameters (amplitude, peak position, and peak width) as a function of temperature for the experimentally observable phonon modes $B_2^{(1)}$ to $B_2^{(9)}$.

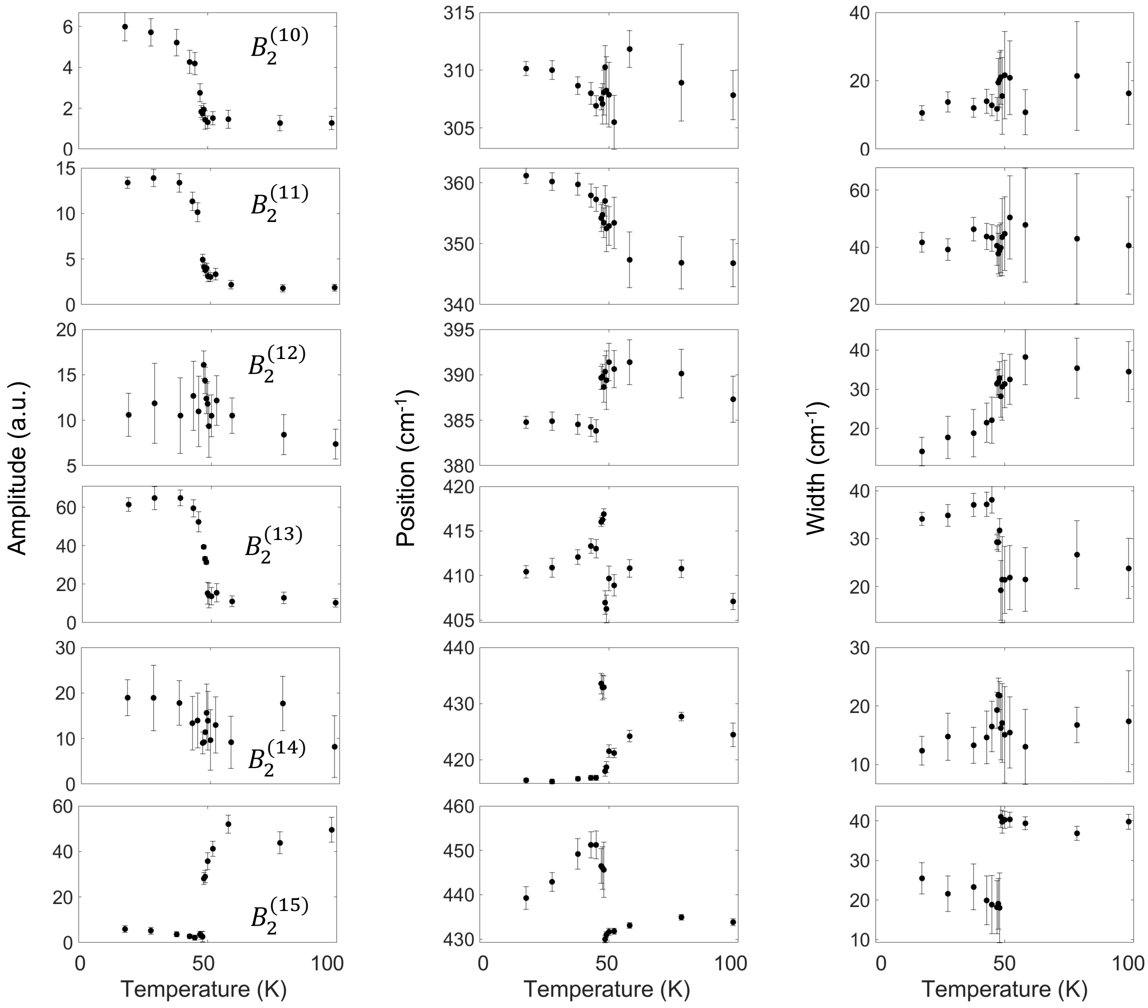

Supplementary Figure 10. Temperature dependent fitting results of $B_2^{(10)}$ to $B_2^{(15)}$. Fitting parameters (amplitude, peak position, and peak width) as a function of temperature for experimental observable phonon modes $B_2^{(10)}$ to $B_2^{(15)}$.

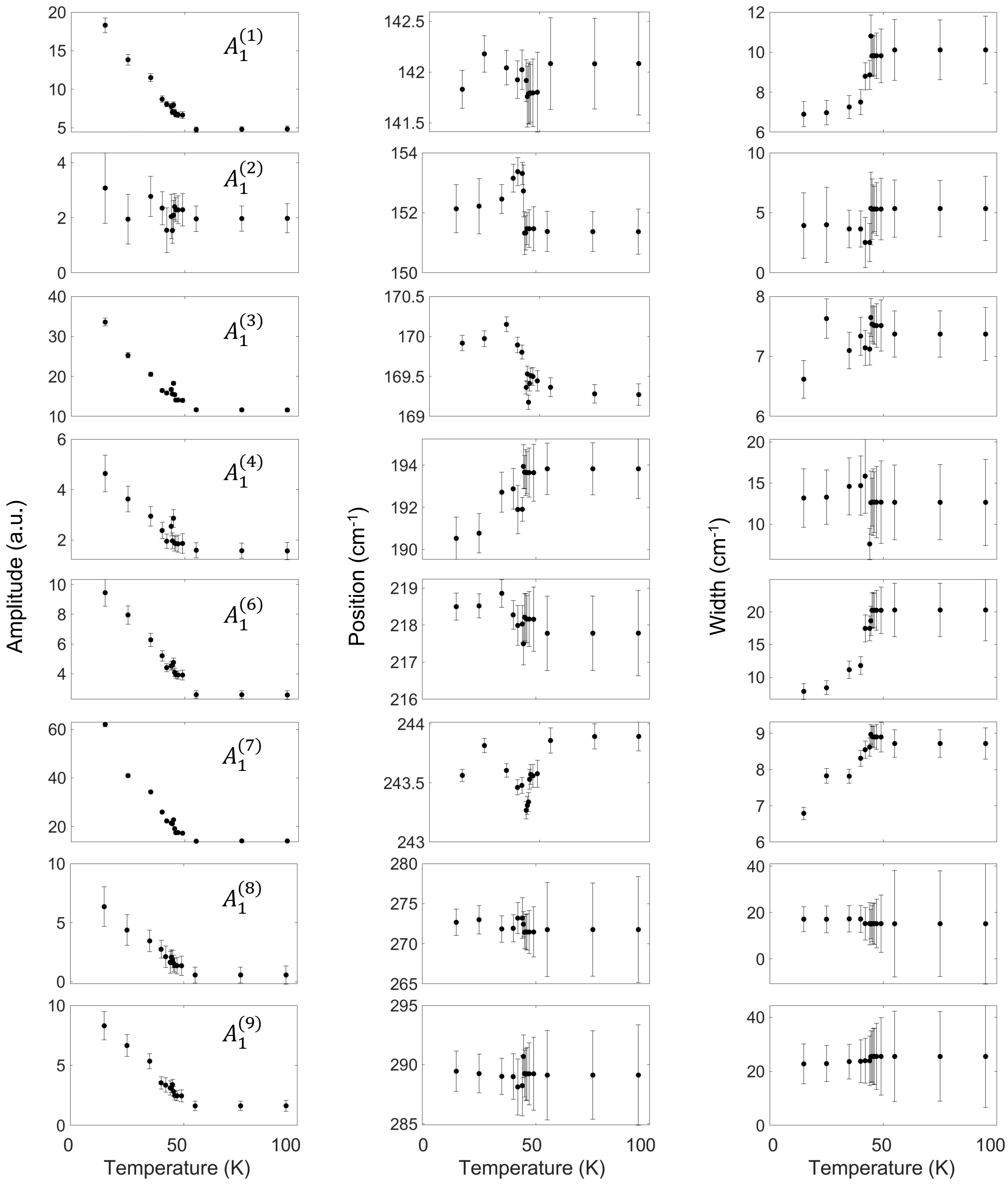

Supplementary Figure 11. Temperature dependent fitting results of $A_1^{(1)}$ to $A_1^{(9)}$. Fitting parameters (amplitude, peak position, and peak width) as a function of temperature for experimental observable phonon modes $A_1^{(1)}$ to $A_1^{(9)}$.

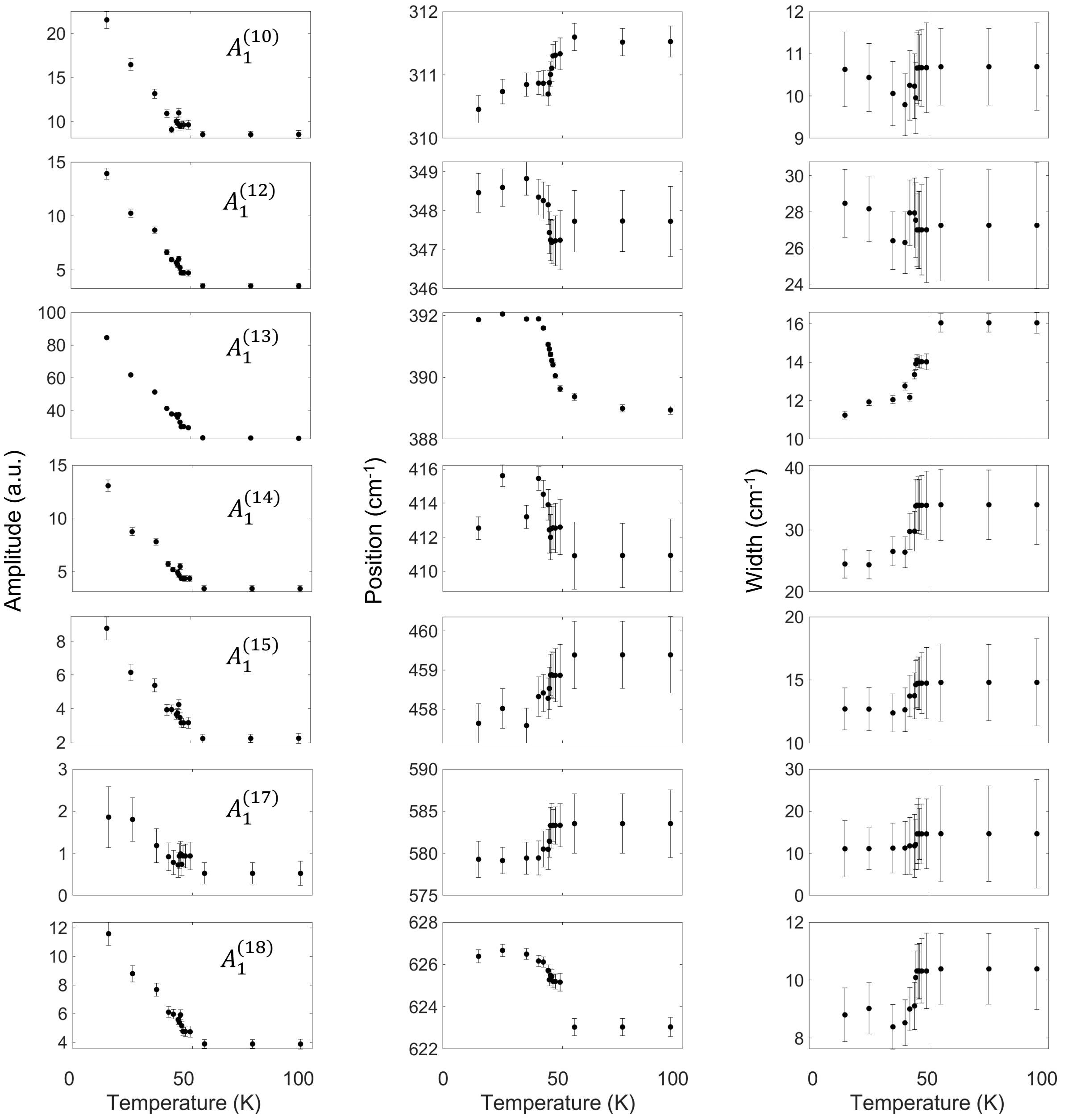

Supplementary Figure 12. Temperature dependent fitting results of $A_1^{(10)}$ to $A_1^{(18)}$. Fitting parameters (amplitude, peak position, and peak width) as a function of temperature for experimental observable phonon modes $A_1^{(10)}$ to $A_1^{(18)}$.

Supplementary Note 8: Joint Density of States results from the DFT calculations

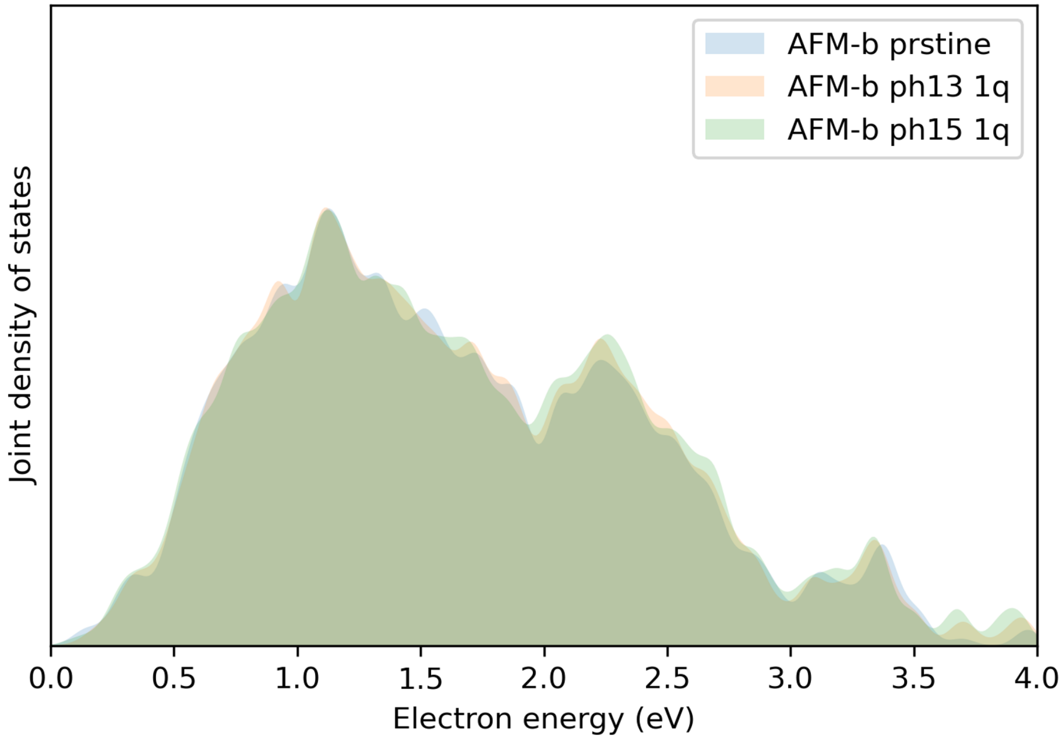

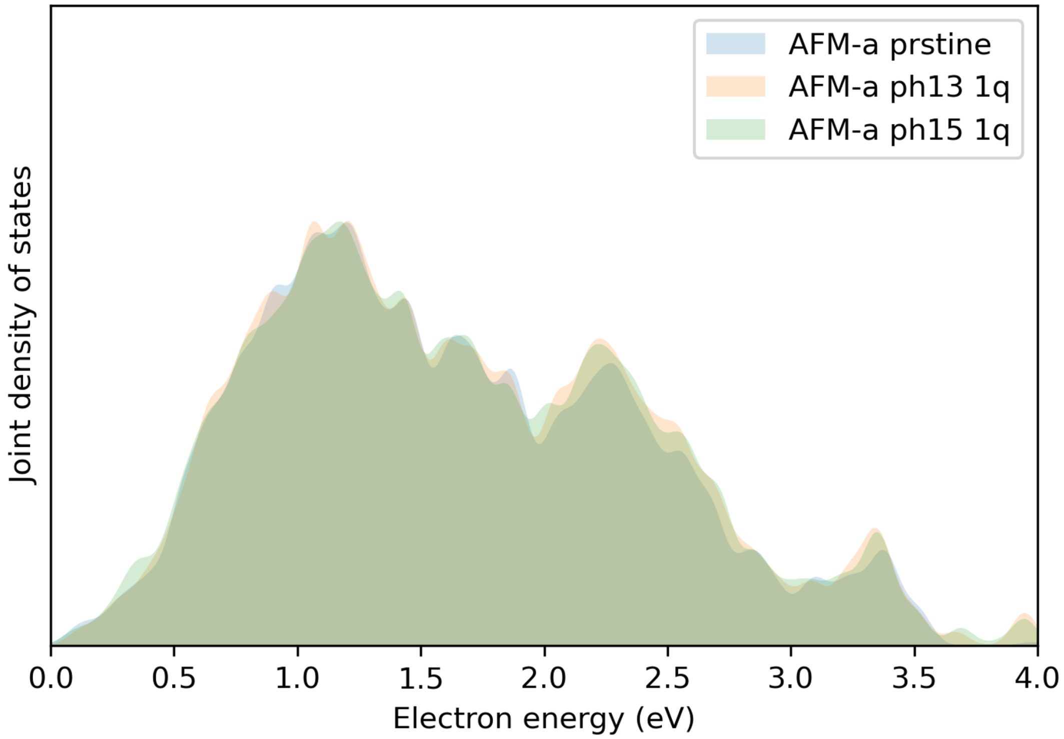

Supplementary Figure 13. Joint density of states calculation results. Joint density of states calculated from all possible optical transitions between -2eV to 2eV in (up) AFM-*b* electronic band structures and (down) AFM-*a* electronic band structures. The phonon perturbed Joint Density of States are very close to those of unperturbed state. This indicates that the Fermi-Golden rule is largely not affected by the phonon perturbation and that the change in FWHM across T_c is mainly from a change in the modulus of the mode specific electron phonon coupling (EPC) matrix.

Supplementary Note 9: Detailed analysis of B_2^P and B_2^M phonons

Considering the importance of the B_2^P and B_2^M phonons in changing the electronic properties of $Ca_3Ru_2O_7$, we list the detailed eigen vector of the two modes in **Supplementary Table 5 and 6**. The name convention for atoms is illustrated in **Supplementary Fig. 14**. The unit cell $Ca_3Ru_2O_7$ hosts two RuO_6 octahedra that changes in different way when perturbed by B_2 phonons. To highlight that difference, we label one of them Octahedra 1 (in blue) and the other one Octahedra 2 (in red).

Supplementary Table 5. The 1 Q (Å$\sqrt{amu}$) eigen vector of B_2^P phonon with initial atomic position of moving atoms respectively.

Octahedral	Atom labels	Atomic position (Å)	Displacement vector (Å)
Oct_1	Ru	[1.369, 4.232, 7.873]	[-5.32e-3, 9.6e-4, 1.93e-3]
Oct_1	O(1)	[0.961, 4.104, 5.916]	[2.75e-2, -2.64e-2, -1.08e-2]
Oct_1	O(2)	[1.853, 4.362, 9.808]	[2.42e-2, 3.16e-2, 0]
Oct_1	O(3)_1	[-0.291, 5.353, 8.239]	[6.03e-2, 6.08e-2, -2.29e-2]
Oct_1	O(3)_2	[0.291, 2.533, 8.239]	[6.03e-2, -6.08e-2, 2.29e-2]
Oct_1	O(4)_1	[2.438, 5.901, 7.498]	[-5.30e-2, 5.08e-2, 3.87e-3]
Oct_1, Oct_2	O(4)_2	[2.990, 3.082, 7.498]	[-5.30e-2, -5.08e-2, -3.87e-3]
Oct_2	Ru	[4.059, 1.413, 7.873]	[-5.32e-3, -9.6e-4, -1.93e-3]
Oct_2	O(1)	[4.467, 1.285, 5.916]	[2.75e-2, 2.64e-2, 1.08e-2]
Oct_2	O(2)	[3.575, 1.543, 9.808]	[2.42e-2, -3.16e-2, 0]
Oct_2	O(3)_1	[5.137, -0.286, 8.239]	[6.03e-2, 6.08e-2, -2.29e-2]
Oct_2	O(3)_2	[5.720, 2.533, 8.239]	[6.03e-2, -6.08e-2, 2.29e-2]
Oct_2	O(4)_1	[2.438, 0.263, 7.498]	[-5.30e-2, 5.08e-2, 3.87e-3]

Supplementary Table 6. The 1 Q (Å$\sqrt{\text{amu}}$) eigen vector of B_2^M phonon with initial atomic position of moving atoms respectively.

Octahedral	Atom labels	Atomic position (Å)	Displacement vector (Å)
Oct_1	O(1)	[0.961, 4.104, 5.916]	[2.24e-2, -5.38e-2, -1.41e-2]
Oct_1	O(2)	[1.853, 4.362, 9.808]	[7.62e-2, -9.23e-2, 0]
Oct_1	O(3)_1	[-0.291, 5.353, 8.239]	[-2.73e-2, -2.25e-2, 2.55e-2]
Oct_1	O(3)_2	[0.291, 2.533, 8.239]	[-2.73e-2, 2.25e-2, -2.55e-2]
Oct_1	O(4)_1	[2.438, 5.901, 7.498]	[-2.49e-2, 8.33e-3, -3.27-2]
Oct_1, Oct_2	O(4)_2	[2.990, 3.082, 7.498]	[-2.49e-2, -8.33e-3, 3.27-2]
Oct_2	Ru	[4.059, 1.413, 7.873]	[3.44e-4, -1.25e-2, -1.19e-3]
Oct_2	O(1)	[4.467, 1.285, 5.916]	[2.24e-2, 5.38e-2, 1.41e-2]
Oct_2	O(2)	[3.575, 1.543, 9.808]	[7.62e-2, 9.23e-2, 0]
Oct_2	O(3)_1	[5.137, -0.286, 8.239]	[-2.73e-2, -2.25e-2, 2.55e-2]
Oct_2	O(3)_2	[5.720, 2.533, 8.239]	[-2.73e-2, 2.24e-2, -2.55e-2]
Oct_2	O(4)_1	[2.438, 0.263, 7.498]	[-2.49e-2, 8.33e-2, -3.27e-2]

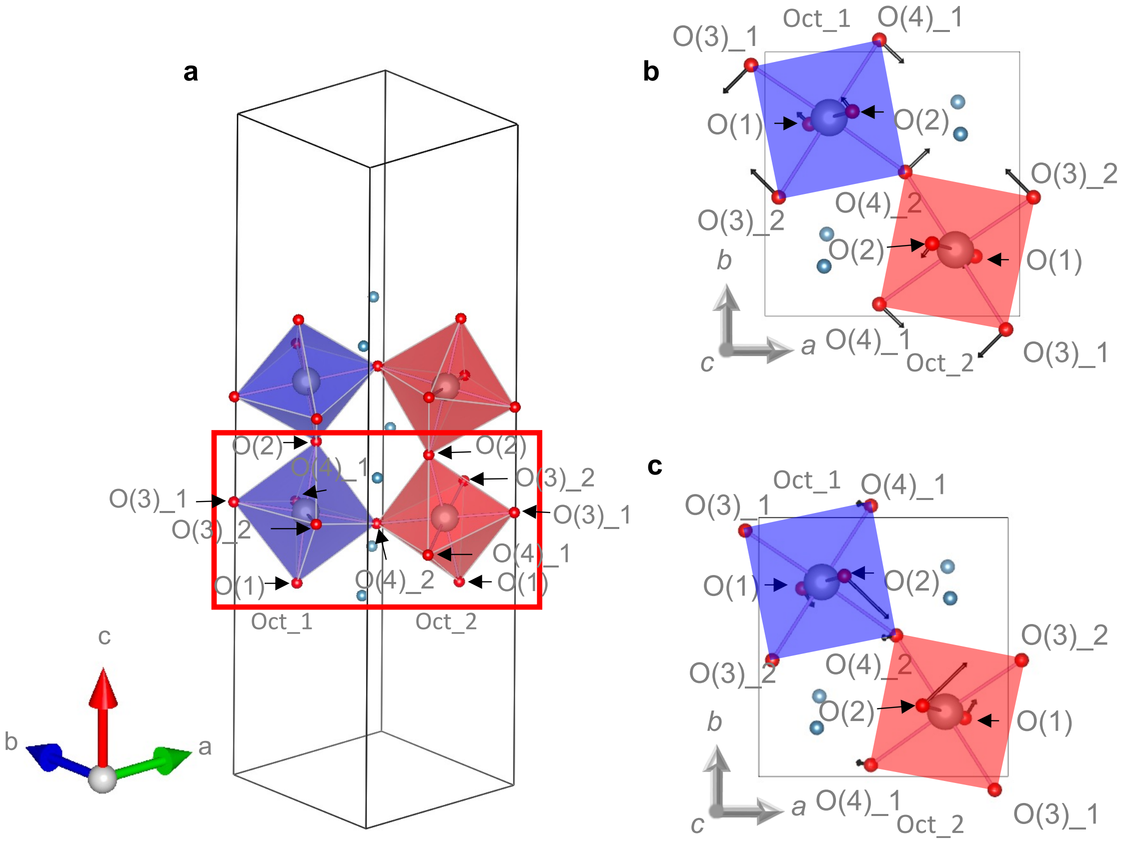

Supplementary Figure 14. Illustration of atom labels. (a) Tilted view of the bi-layer atomic structure of $Ca_3Ru_2O_7$. The red rectangular shows the two octahedra that is selected to plot in (b) and (c). (b) The top view of atomic structure and the black arrows are the eigen vectors eigen vector of B_2^P. (c) The top view of atomic structure and the black arrows are the eigen vectors of B_2^P. The blue shade indicates octahedra 1, and the red shade indicates octahedra 2.

The overall electronic property perturbed by phonons can be explained by the average change in $d_\perp/d_{//}$ and in-plane bond angle (θ) of two types of octahedra as shown in **Fig. 4**. The B_2^P and B_2^M phonons would asymmetrically change the $d_\perp/d_{//}$ and θ of individual Octahedron while the average change of Octahedra 1 and Octahedra 2 is symmetric (**Supplementary Fig.15)**. The detailed analysis of phonon perturbed Octahedra 1 and Octahedra 2 shows important differences

between B_2^P and B_2^M phonons. B_2^P phonon of 1 Q decreases/increases the in-plane hopping integral along $[1\ \bar{1}\ 0]/[1\ 1\ 0]$ crystallographic direction due to a synchronized in-plane bond angle and bound length change that affects the hopping integral in the same way (**Supplementary Fig.16a**). On the other hand, B_2^M phonon cannot modulate hopping parameters due to competing effects from bond length and bond angle change (**Supplementary Fig.16b**).

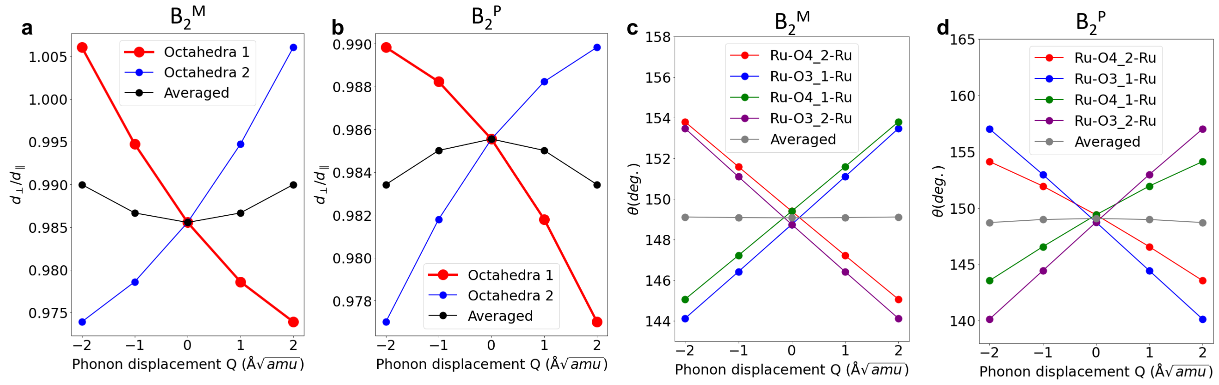

Supplementary Figure 15. d ratio and in-plane bond angles of octahedra 1 and 2 modulated by $\boldsymbol{B_2^M}$ and $\boldsymbol{B_2^P}$ phonons. The ratio of RuO_6 octahedra cage apical Ru-O bond length ($d_\perp$) over in-plane bond length ($d_\parallel$) calculated at two neighboring octahedra modulated by (a) B_2^M and (b) B_2^P phonon. Ru-O-Ru in-plane bond angles modulated by (c) B_2^M and (d) B_2^P phonon. The name convention agrees with illustration in **Supplementary Fig. 14**.

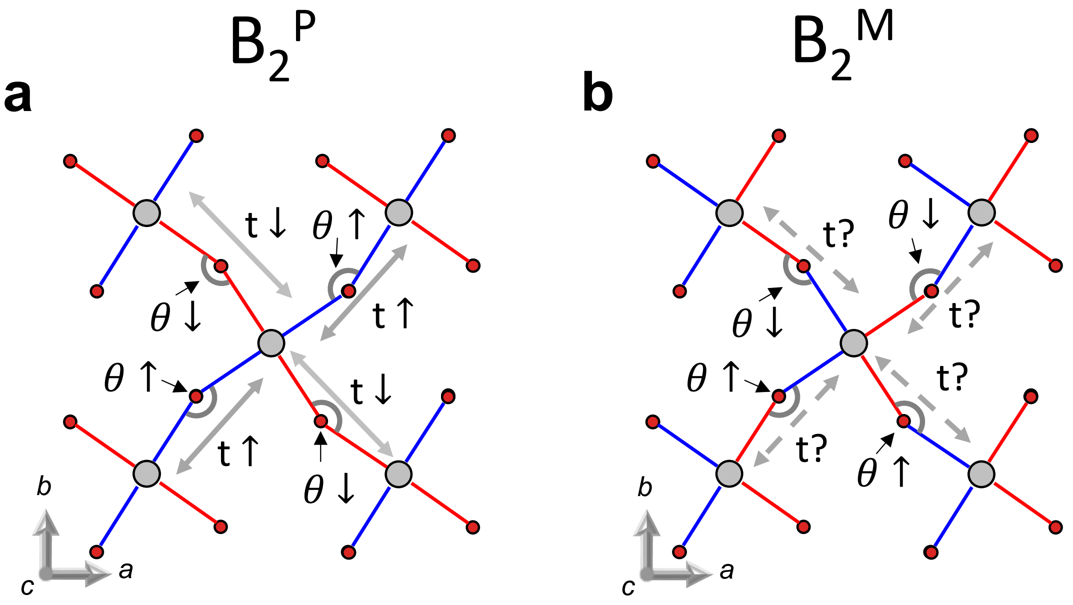

Supplementary Figure 16. In-plane hopping integral modulated by B_2^{P} and B_2^{M} phonon. (a) The real-space Ru-O bond length change (color code: red means increase, and blue means decrease) and in plane Ru-O-Ru bond angle change (θ ↑ means angle increase, and θ ↓ means angle decrease) after 1Q B_2^{P} phonon modulation. This phonon increases the hopping integral t either along the [1 1 0] or along the [1 $\bar{1}$ 0] crystallographic direction. (b) The real-space Ru-O bond length change (color code: red means increase, and blue means decrease) and in plane Ru-O-Ru bond angle change (θ ↑ means angle increase, and θ ↓ means angle decrease) after 1Q B_2^M phonon modulation. The change in the hopping integral t along any of the directions is not monotonic due to competing effects from bond length changes and θ changes after 1Q B_2^M phonon displacement.

Supplementary Note 10: Raman Vertex

The Raman Vertex of A_1 (with symmetry of x^2 or y^2, see **Table 1**) is a linear combination of $\gamma_{x^2+y^2}$ and $\gamma_{x^2-y^2}$. The Raman Vertex of B_2 (with symmetry of xy, see **Table 1**) is γ_{xy}. The Raman Vertex is a form factor for electronic Raman that includes symmetry consideration described as following:

$$\rho_u(\omega) = \sum_{\mathbf{k}} \gamma_u(\mathbf{k}) n(\mathbf{k}) = \sum_{\boldsymbol{k}} \gamma_u\,(\mathbf{k}) c^{\dagger}_{\mathbf{k}\sigma} c_{\mathbf{k}\sigma}$$

$c^{\dagger}_{\boldsymbol{k}\sigma}$ and $c_{\boldsymbol{k}\sigma}$ are creation and annihilation operators of an electron with momentum **k** and spin σ. The Raman Vertex is then defined as:

$$\gamma_u\,(\mathbf{k}) = \gamma_{\alpha,\beta}\,(\mathbf{k}) e_i^{\alpha} e_s^{\beta}$$

Here if we ignore the screening effects, the free-electron Raman Vertex only depends on band dispersions $E_{\mathbf{k}}$:

$$\gamma_{\alpha,\beta}\,(\mathbf{k}) = \frac{1}{\hbar^2} \frac{\partial^2 E_{\mathbf{k}}}{\partial k_{\alpha} \partial k_{\beta}}$$

With the selection rule implemented by harmonic basis functions[11,12], we get the following expression for γ_{xy}, $\gamma_{x^2+y^2}$ and $\gamma_{x^2-y^2}$:

$$\gamma_{xy} = \frac{1}{\hbar^2} \frac{\partial^2 E_{\mathbf{k}}}{\partial k_x \partial k_y} \propto \sin\,(k_x a) \sin\,(k_y b)$$

$$\gamma_{x^2+y^2} = \frac{1}{2\hbar^2} \left(\frac{\partial^2 E_{\mathbf{k}}}{\partial k_x^2} + \frac{\partial^2 E_{\mathbf{k}}}{\partial k_y^2} \right) \propto \cos(k_x a) + \cos\,(k_y b)$$

$$\gamma_{x^2-y^2} = \frac{1}{2\hbar^2} \left(\frac{\partial^2 E_{\mathbf{k}}}{\partial k_x^2} - \frac{\partial^2 E_{\mathbf{k}}}{\partial k_y^2} \right) \propto \cos(k_x a) - \cos\,(k_y b)$$

Supplementary Note 11: Hund's Rule description

The density-density interactions between electrons can be categorized into the following three conditions:

1. Electrons with opposite spins in the same orbital have a correlation energy of U.
2. Electrons with opposite spins in different orbitals have a correlation energy of U'.
3. Electrons with parallel spins in different orbitals have a correlation energy of $U' - J$.

Under the approximation where the screened coulomb interaction is spherical symmetry, which is often a reasonable approximation, one can derive that $U' = U - 2J$.[13] Ru^{4+} hosts 4 electrons in the d orbital in the following configuration: $d_{xy}^2 d_{xz}^1 d_{yz}^1$. Now consider the following transition: $d^4 + d^4 \rightarrow d^3 + d^5$. Here we only consider the situation described in the main text in **Fig. 5g**, namely d^3 is $d_{xy}^1 d_{xz}^1 d_{yz}^1$ and d^5 is $d_{xy}^1 d_{xz}^2 d_{yz}^1$ or $d_{xy}^1 d_{xz}^1 d_{yz}^2$ configurations. Further, we only consider the electron coupling energy and assume that the d orbitals are close enough to be considered as degenerate. Before the transition, we have the following correlation energy: $2 * (U + 3 * (U' - J) + 2 * U')$. After the transition, we have: $[3 * (U' - J)] + [2 * U + 4 * (U' - J) + 4 * U']$. If we calculate the energy difference before and after the transition, we get $U - 3J$.

Supplementary Note 12: Electronic Raman Background related analysis

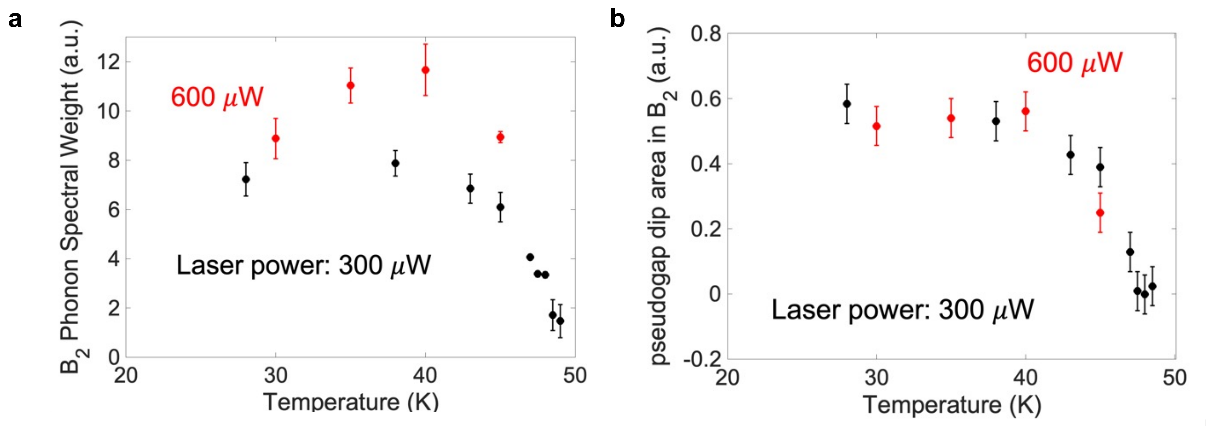

Supplementary Figure 17. Temperature dependence of B_2^P phonon amplitude and pseudogap dip area. (a) Temperature dependence of pseudo gap dip area in $\Delta\chi''_{B_2}(\omega, T)$ between 120 cm^{-1} and 400 cm^{-1} at 300μW and 600μW incident laser power. The dip of B_2^P phonon intensity with 600 μW is likely due to artifacts since this change in intensity has also been observed in other B_2 phonons under the same condition (see **Supplementary Fig. 18a-b**). (b) Temperature dependence of $B_2^{(13)}$ (B_2^P) spectral weight at 300μW and 600μW incident laser power.

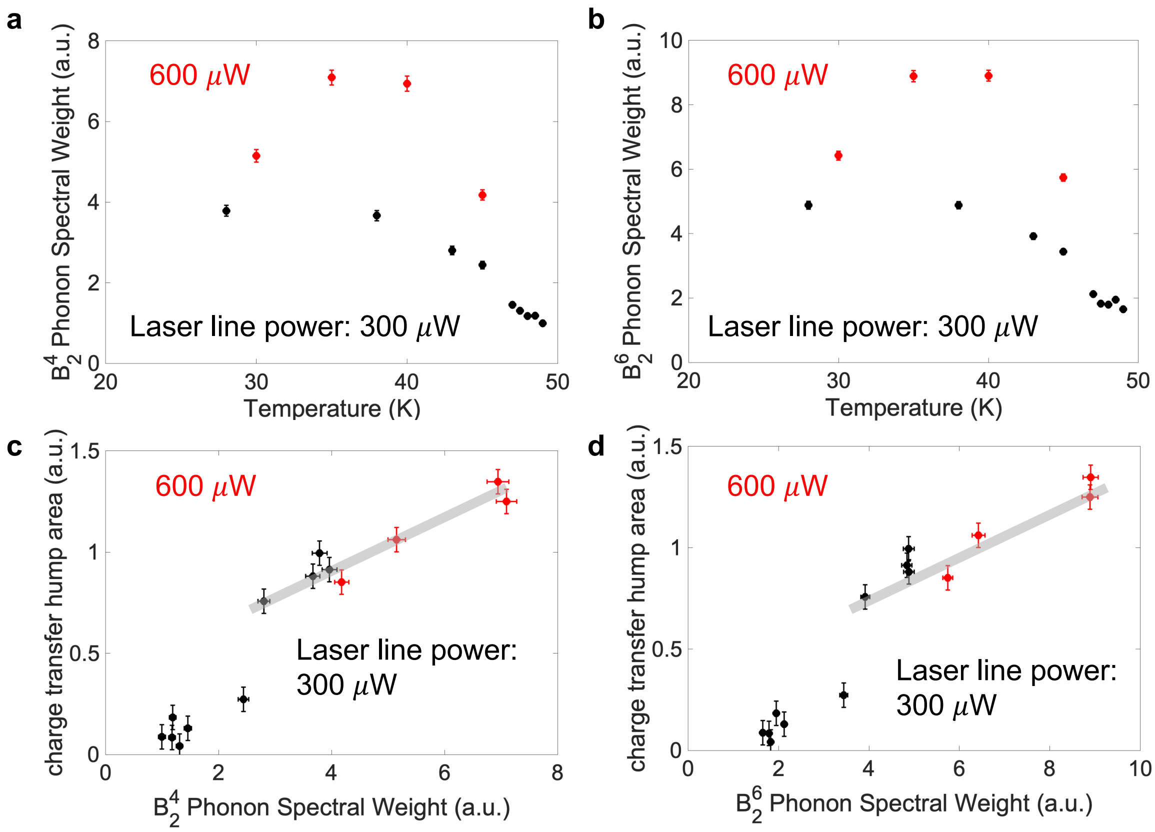

Supplementary Figure 18. Temperature dependence of $B_2^{(4)}$ and $B_2^{(6)}$ and their correlation with charge transfer hump area. Temperature dependence of (a) $B_2^{(4)}$ and (b) $B_2^{(6)}$ spectral weight at 300μW and 600μW incident laser power. Correlation between (c) $B_2^{(4)}$ and (d) $B_2^{(6)}$ spectral weight and charge transfer Raman response at 300μW and 600μW incident laser power.

Here we plot the next two strongest peak in B_2 geometry besides $B_2^{(13)}$, which are $B_2^{(4)}$ around 207 cm^{-1} and $B_2^{(5)}$ around 220 cm^{-1}. Both peaks show correlation with the intersite charge transfer feature, which is the 'hump' feature in the background. Thus, we cannot isolate a specific phonon responsible for mediating inter-site charge transfer. However, the $B_2^{(4)}$ and $B_2^{(6)}$ phonons shows narrow peak width (4 and 6 cm^{-1}) near 48K compared to ~40 cm^{-1} peak width in $B_2^{(13)}$ (also known as B_2^{P}). From Eq 2 in the main text, it is unlikely the $B_2^{(4)}$ and $B_2^{(6)}$ phonons

couple to electronic structure strongly. The overall similarity in temperature dependence of Raman intensity among the B_2 phonons is likely a result of continuous bond length and bond angle changes below T_c although the lattice volume is largely unchanged.[2,14]

Supplementary Note 13: Optical Selection Rule on d-d transition between neighboring Ru sites

The *B*2 phonon mediates intersite charge transfers between neighboring Ru atoms. From the illustration in **Fig. 5g**, there are two steps for the intersite charge transfer:

1) The electron hops from O p_z orbital (p_z^2 has a A_1 symmetry) to Ru $d_{xz/yz}$ orbital (octahedra 1, $d_{xy}^2 d_{xz}^1 d_{yz}^1$ has a B_2 symmetry). This transition can be assisted via the *x* component of transition moment operator $\langle\psi_e'|\mu_x|\psi_e\rangle = \langle B_2|B_2|A_1\rangle = A_1$. Next, the electron from neighboring Ru d_{xy} (octahedra 2, $d_{xy}^2 d_{xz}^1 d_{yz}^1$ has a B_2 symmetry) hops to O p_z orbital (p_z^1 has a B_1 symmetry). This transition is optically forbidden but can be facilitated by the B_2 phonons via $\langle\psi_e'\psi_v'|\mu_z|\psi_e\psi_v\rangle = \langle B_2B_1|B_1|B_2A_1\rangle = A_1$. This can explain the Raman background increase observed only in the B_2 geometry shown in **Fig. 5a-d**.

2) The back hopping transition starts with the electron in the O p_z (p_z^2 has a A_1 symmetry) hopping to Ru $d_{xz/yz}$ (octahedra 2, $d_{xy}^1 d_{xz}^1 d_{yz}^1$ has a A_1 symmetry). This transition can be assisted via the *y* component of transition moment operator $\langle\psi_e'|\mu_y|\psi_e\rangle = \langle A_1|A_1|A_1\rangle = A_1$. Next, the electron in Ru $d_{xz/yz}$ (octahedra 1 $d_{xy}^2 d_{xz}^2 d_{yz}^1$ has a B_1 symmetry; $d_{xy}^2 d_{xz}^1 d_{yz}^2$ has a A_2 symmetry) hopping to O p_z (p_z^1 has a B_1 symmetry). This transition can either be assisted via the *y* component of transition moment operator for the B_1 to B_1 case: $\langle\psi_e'|\mu_y|\psi_e\rangle = \langle B_1|A_1|B_1\rangle = A_1$, or assisted by x component for A_2 to B_1 case: $\langle\psi_e'|\mu_x|\psi_e\rangle = \langle A_2|B_2|B_1\rangle = A_1$. Thus, the transitions in step 2 are all optical selection rules allowed.

Supplementary Note 14: Lack of static charge density wave phase in $Ca_3Ru_2O_7$ from structural evidence

The recent X-ray PDF study[14] of $Ca_3Ru_2O_7$ reports the Ru-O bond length with an error bar of 0.01 Å. The reported structure distortion that induces the static charge density wave phase by the DFT study[15] result in a bond length difference of the neighboring in-plane Ru-O bond as large as 0.07Å, which one should have been able to be resolve in the X-ray study. Nonetheless, the experimental PDF study reported that $Bb2_1m$ space group shows a reasonable fit down to 10K, thus most likely ruling out the $Pn2_1a$ phase.

Here, we provide an alternative explanation for the ground state of $Ca_3Ru_2O_7$: the structure change revealed by X-ray diffraction across 48K includes a splitting of in-plane angles (See Fig. 7a in reference 14) and a reduction in the $d_{\perp}/d_{//}$ (See Fig. 8a in reference 14). The following discussion follows the labels in **Supplementary Fig. 19**. As temperature is lowered below 48K, the Ru-O(3)-Ru bond angle (β_1) decreases, which would decrease the hopping integral. However, the Ru-O(3) bond length decreases (R_1), which would increase the hopping integral. A similar argument made for β_2 and R_2, results in bond angle (β_2) increases the hopping integral and the bond length (R_2) reduces the hopping integral. Due to a lack of electron hopping promotion from structural distortion at low temperature, the density wave distortion is not formed; however, due to the reduction in $d_{\perp}/d_{//}$, the Fermi-surface is partially gapped. In contrast, the B_2^{P} can coherently modulate the hopping integral along $(1\ \bar{1}\ 0)$ direction (**Supplementary Fig.16 a**), which indicates driving B_2^{P} can potentially distort the structure towards a charge density wave modulation.

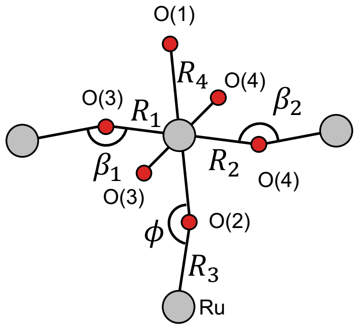

Supplementary Figure 19. Temperature-dependence of bond length and bond angles in $Ca_3Ru_2O_7$. Illustration of the Ru and O atoms, labels of bond lengths between Ru and O atoms, and labels of the bond angle of O-Ru-O consistent with Fig. 7a and 7b in reference 14

The DFT study[15] predicting $Pn2_1a$ is nonetheless potentially insightful in that this phase exhibits a bifurcation of the neighboring octahedral volume. Excitation of B_2 phonons can modulate the neighboring octahedra volumes in a similar manner (see **Supplementary Fig. 20**, using B_2^{P} as an example, but the phenomenon is universal to all B_2 modes). This makes the excitation of B_2 phonons as a potential pathway to stabilize a dynamic density wave phase in $Ca_3Ru_2O_7$.

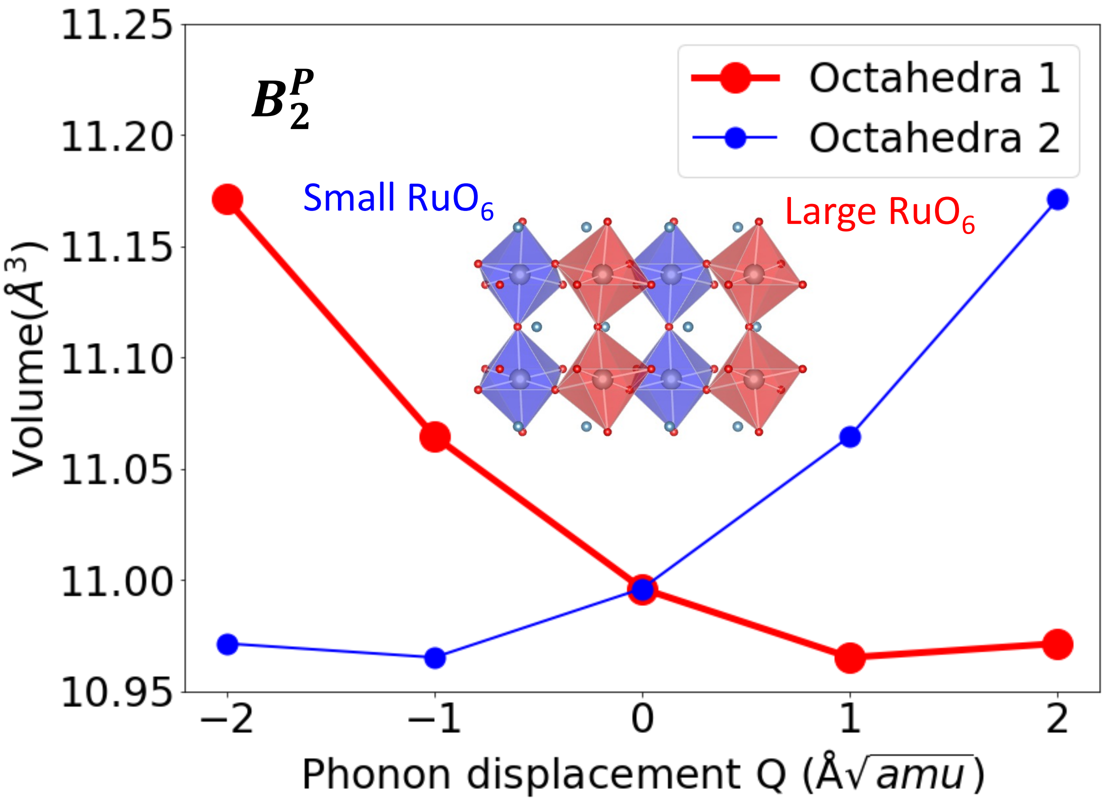

Supplementary Figure 20. Volume perturbation by B_2^{P} phonon mode. The volume of Octahedra 1 and Octahedra 2 as a function of B_2^{P} phonon displacement.

Supplementary Note 15: Discussion on density wave fluctuation lifetime

The FWHM of the hump feature in **Fig. 5c** can have multiple origins, such as inhomogeneous broadening from defects or various sizes of density wave domains. This makes accessing the intrinsic lifetime of the density wave fluctuation challenging. As a very rough estimate, the rising edge of the hump (see **Supplementary Fig. 21a**) can be well described by a Gaussian fitting which is estimated to be 135cm^{-1} at 18K, which translates to around 250fs in coherence lifetime. The FWHM of density wave fluctuation feature increases (and hence the lifetime decreases) as the temperature increases (**Supplementary Fig. 21b**), which has the same trend as the energy of the B_2^{P} phonon (**Supplementary Fig. 21c**). The frequency of the B_2^{P} phonon has a temporal period of ~83fs at 18K, which is a third of the lifetime of the density wave fluctuation. This suggests that these phonons can indeed mediate the density wave fluctuations on similar timescales. To further study the lifetime of density wave fluctuations, we suggest future experiments such as ultrafast pump probe to accurately characterize both the temporal and spatial coherence of the density wave fluctuation.

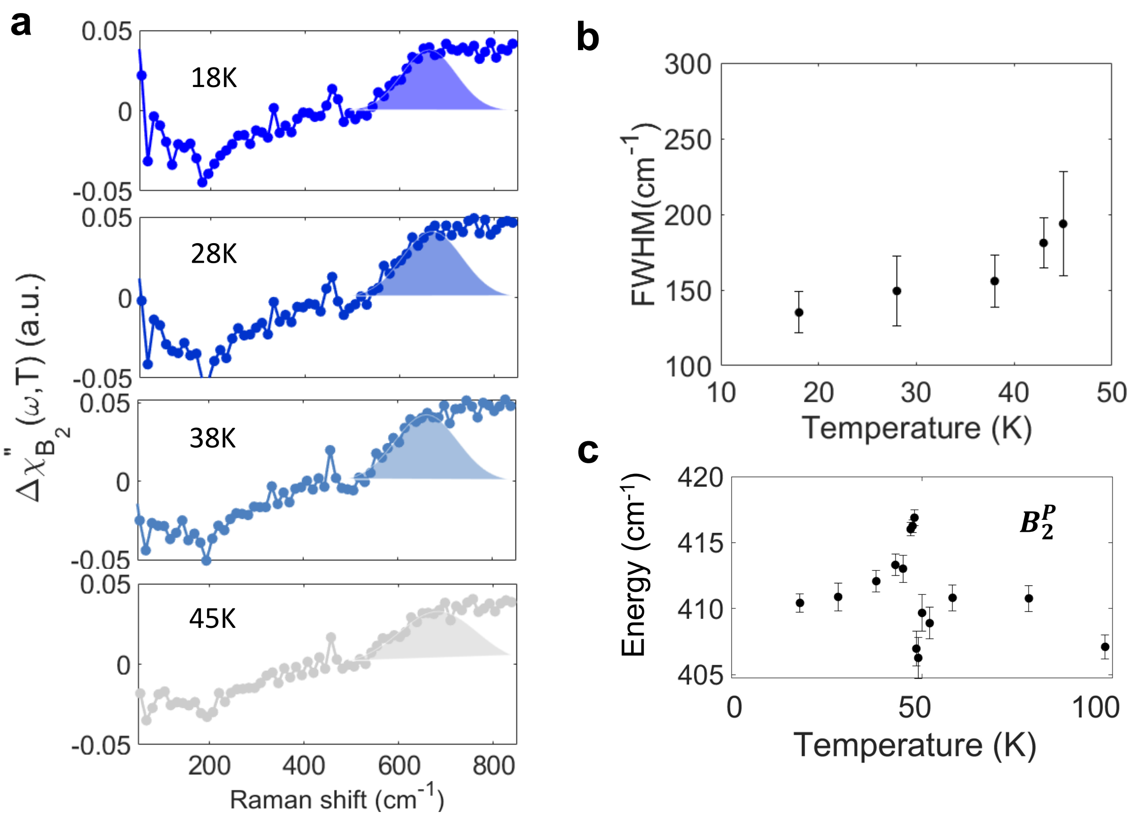

Supplementary Figure 21. Temperature-dependence of density wave fluctuation lifetime compared with phonon energy of B_2^{P} phonon. (a) The Gaussian fitting profile (shaded area) overlayed on top of the subtracted electronic Raman response $\Delta\chi''_{B_2}(\omega, T)$ after subtracting the response at 49K (dotted line). The temperature of the collected electronic Raman response is labeled in each graph. (b) The temperature dependence of the FWHM of the fitted Gaussian profile. (c) The phonon energy of the B_2^{P} phonon as a function of the temperature.

Supplementary Note 16: Calculation of laser heating

Here we employed a simple kinetic model to calculate the laser heating effect from the CW laser. Here the laser heating problem can be reduced to 1D heat conduction problem with constant generation:

$$k\frac{d^2T}{dx^2}+q=0$$

Where k is thermal conductivity, q is the heating source, which is $I/(\pi r^2)$, where I is the laser input power and r is the laser focused spot size. The coordinate x here is along the sample thickness direction where $x=0$ is at the surface and $x=x_0$ is the back side of the sample where surface is attached to a heat sink. Thus, x_0 is the sample thickness. The solution of the above equation is:

$$T=-\frac{qx^2}{2k}+Ax+B$$

There are two boundary conditions: (1) at $x=x_0$, the sample is at the cold finger temperature T_0, and (2) at $x=0$, the sample is insulated due to the environment is vacuum. Thus the $\frac{dT}{dx}|_{x=0}=0$. This implies that $A=0$.

To solve for B, we plug in the boundary condition (1). In this study, we reference the work[16] where the thermal conductivity shows small variance below 48K both experimentally and theoretically. We take the thermal conductivity k ≈ 4 W/mK. The focused spot size is estimated to be 3μm in diameter and the thickness of the sample is 0.5mm. The averaged laser heating is found to be 3K using 250 μW laser power. For 1800 gt/mm data, 600 μW laser power was used, which results in a 6K thermal heating.

Supplementary Note 17: Electronic band structure and phonon eigen mode calculated from DFT

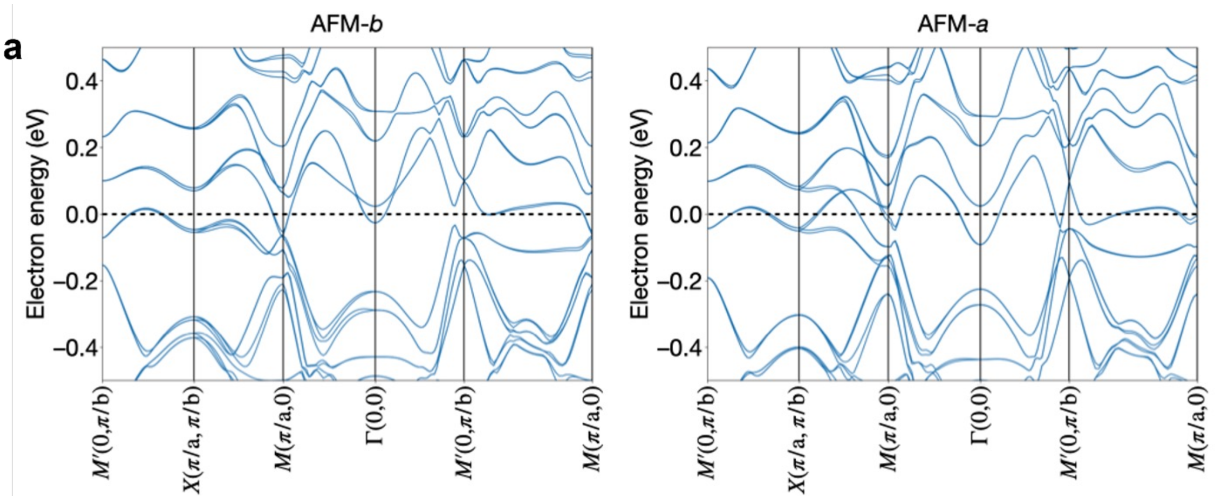

Supplementary Figure 22. DFT calculation of electronic band structures of AFM-*b* and AFM-*a* phases. Electronic band structures of AFM-*b* and AFM-*a* phases using LDA+*U*+SOI at $k_z = 0$. The Fermi level is set to 0 eV.

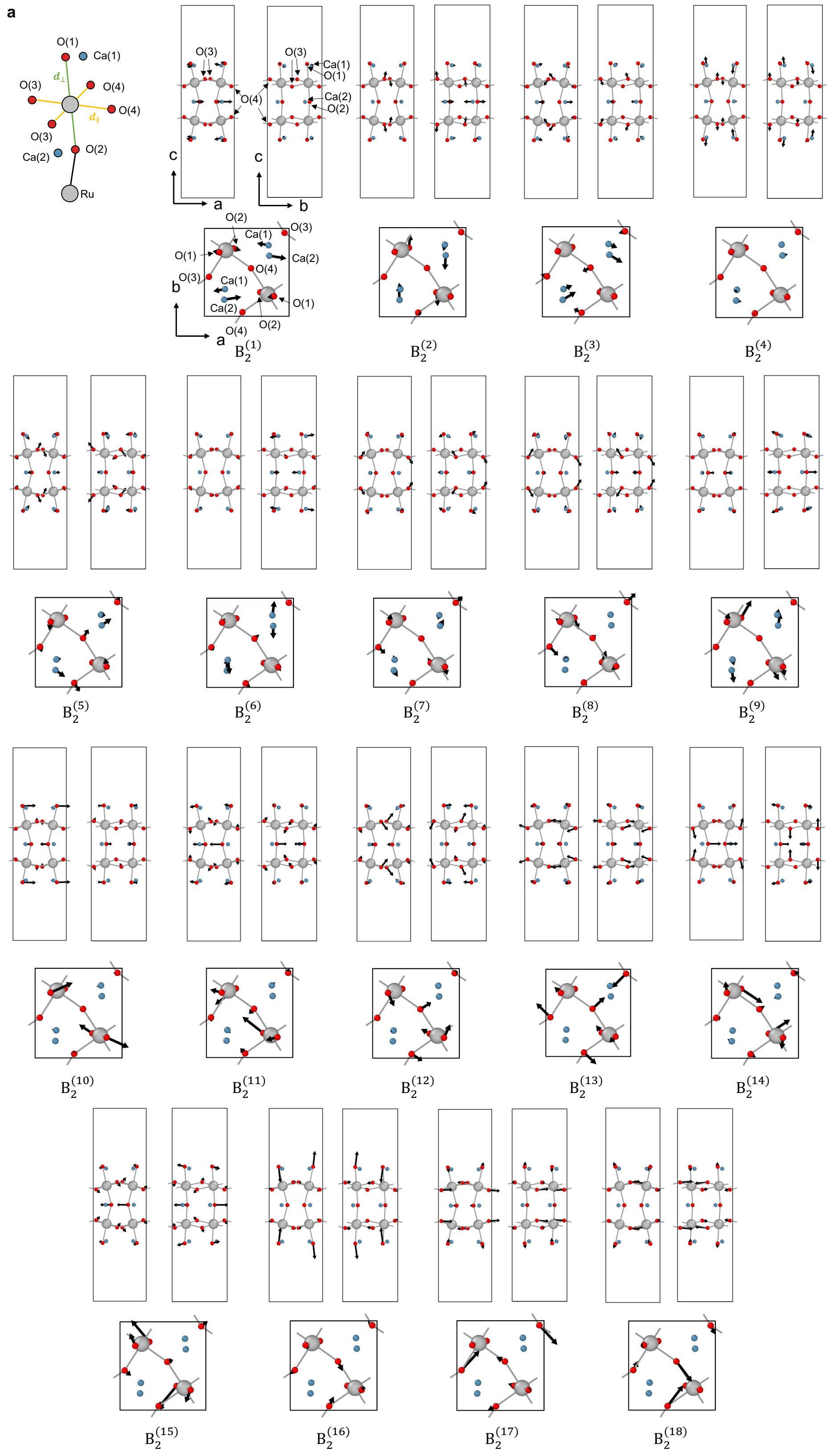
a
O(1)
Ca(1)
O(3)
O(4)
O(4)
O(3)
Ca(2)
O(2)
Ru
O(3)
O(4)
Ca(1)
O(1)
Ca(2)
O(2)
c
a
b
O(2)
Ca(1)
O(3)
O(1)
Ca(2)
O(3)
O(4)
Ca(1)
Ca(2)
O(1)
O(4)
O(2)
$B_2^{(1)}$
$B_2^{(2)}$
$B_2^{(3)}$
$B_2^{(4)}$
$B_2^{(5)}$
$B_2^{(6)}$
$B_2^{(7)}$
$B_2^{(8)}$
$B_2^{(9)}$
$B_2^{(10)}$
$B_2^{(11)}$
$B_2^{(12)}$
$B_2^{(13)}$
$B_2^{(14)}$
$B_2^{(15)}$
$B_2^{(16)}$
$B_2^{(17)}$
$B_2^{(18)}$

b

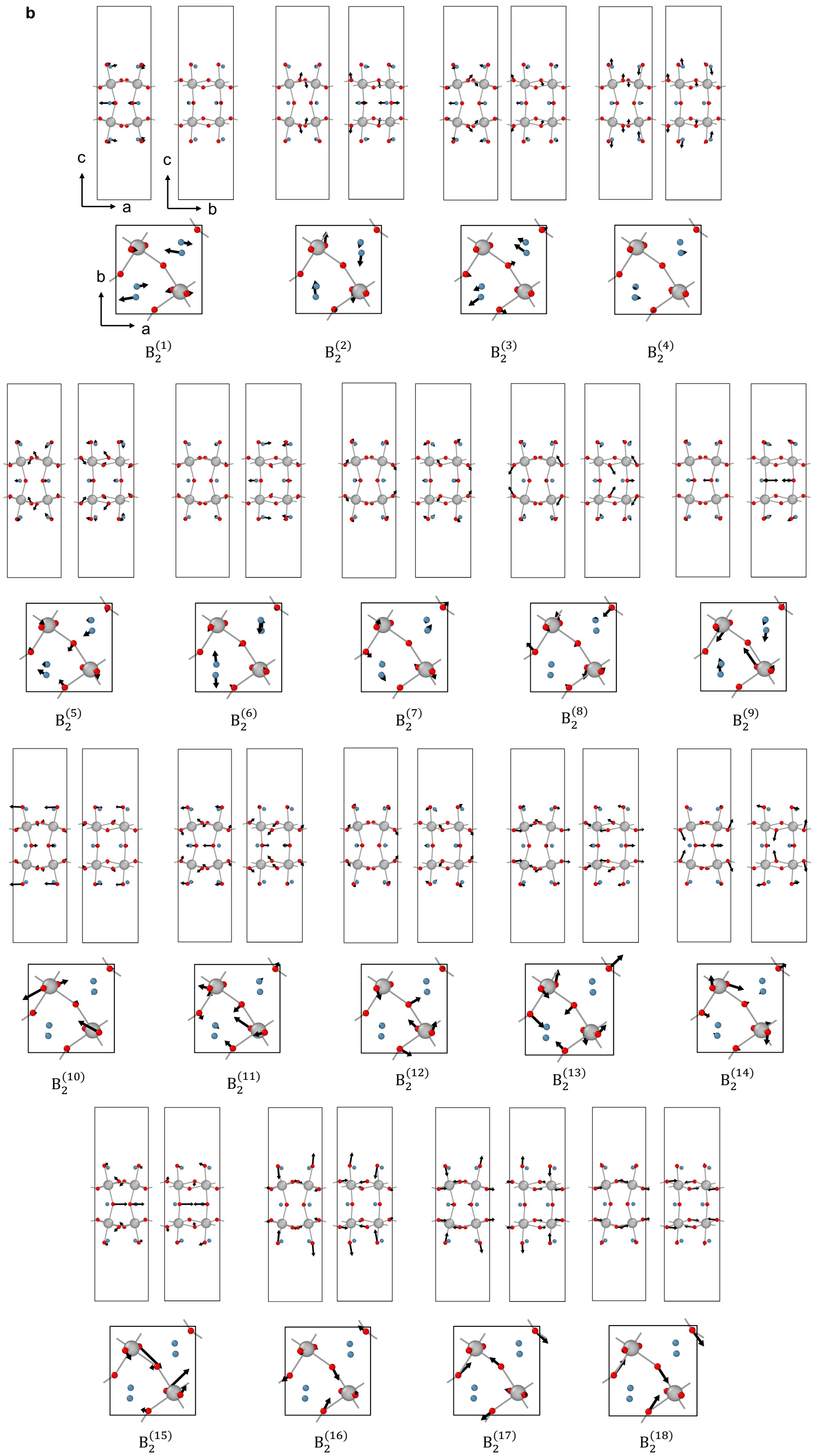
c
a
c
b
b
a
$B_2^{(1)}$
$B_2^{(2)}$
$B_2^{(3)}$
$B_2^{(4)}$
$B_2^{(5)}$
$B_2^{(6)}$
$B_2^{(7)}$
$B_2^{(8)}$
$B_2^{(9)}$
$B_2^{(10)}$
$B_2^{(11)}$
$B_2^{(12)}$
$B_2^{(13)}$
$B_2^{(14)}$
$B_2^{(15)}$
$B_2^{(16)}$
$B_2^{(17)}$
$B_2^{(18)}$

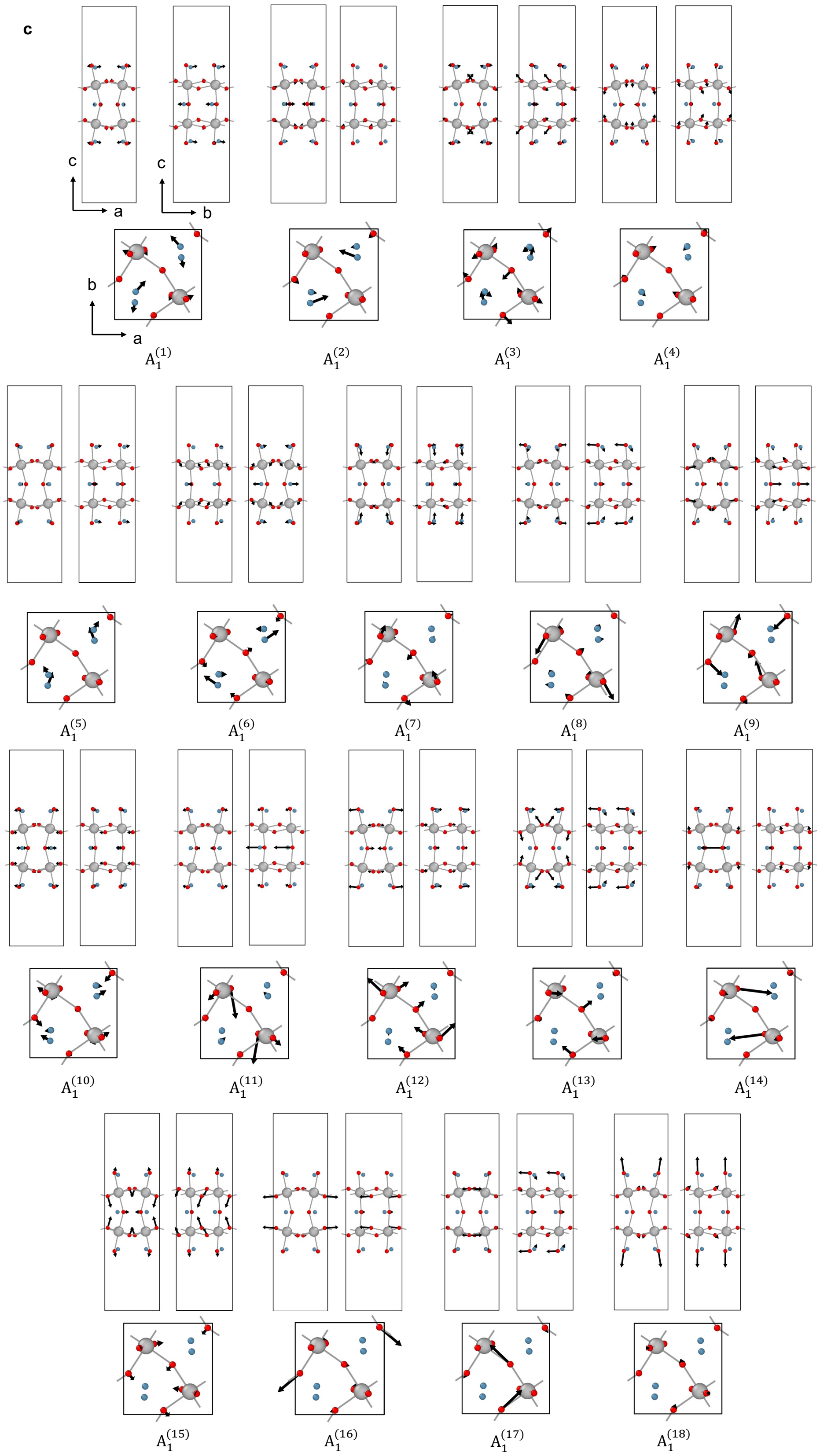

c
c
a
c
b
b
a
$A_1^{(1)}$
$A_1^{(2)}$
$A_1^{(3)}$
$A_1^{(4)}$
$A_1^{(5)}$
$A_1^{(6)}$
$A_1^{(7)}$
$A_1^{(8)}$
$A_1^{(9)}$
$A_1^{(10)}$
$A_1^{(11)}$
$A_1^{(12)}$
$A_1^{(13)}$
$A_1^{(14)}$
$A_1^{(15)}$
$A_1^{(16)}$
$A_1^{(17)}$
$A_1^{(18)}$

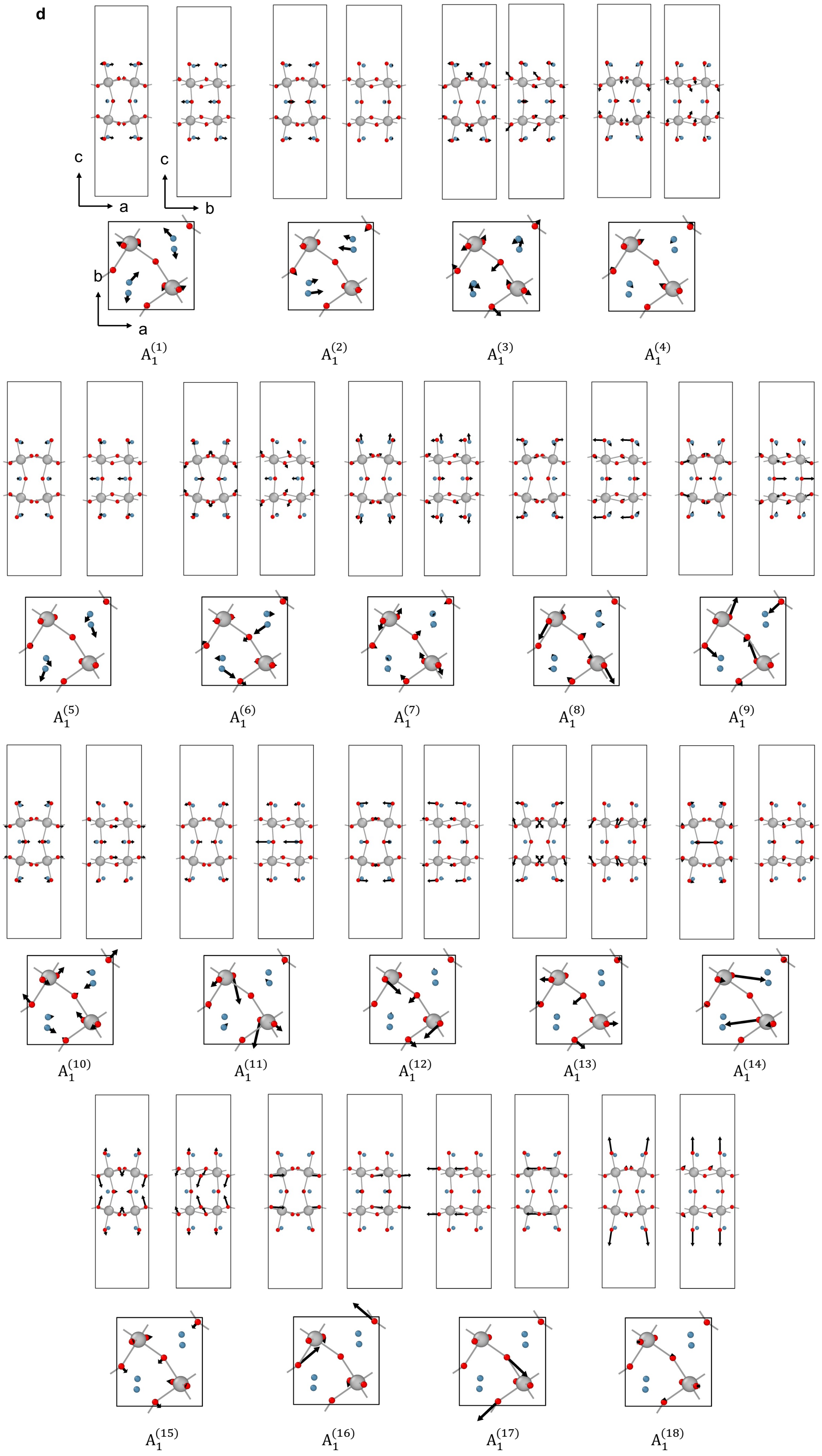
d
c
a
c
b
b
a
$A_1^{(1)}$
$A_1^{(2)}$
$A_1^{(3)}$
$A_1^{(4)}$
$A_1^{(5)}$
$A_1^{(6)}$
$A_1^{(7)}$
$A_1^{(8)}$
$A_1^{(9)}$
$A_1^{(10)}$
$A_1^{(11)}$
$A_1^{(12)}$
$A_1^{(13)}$
$A_1^{(14)}$
$A_1^{(15)}$
$A_1^{(16)}$
$A_1^{(17)}$
$A_1^{(18)}$

Supplementary Figure 23. Eigen modes from VASP frozen phonon calculation. The DFT calculation of phonon Eigen modes: (a) B_2 modes in the AFM-b phase, (b) B_2 modes in the AFM-a phase, (c) A_1 modes in AFM-b phase, (d) A_1 modes in AFM-a phase.

References

1 Iliev, M. N. et al. Raman spectroscopy of Ca3Ru2O7: Phonon line assignment and electron scattering. *Phys Rev B* **71**, (2005).

2 Yoshida, Y. et al. Crystal and magnetic structure of Ca3Ru2O7. *Phys Rev B* **72** (2005).

3 Liu, H. L., Yoon, S., Cooper, S. L., Cao, G. & Crow, J. E. Raman-scattering study of the charge and spin dynamics of the layered ruthenium oxide Ca3Ru2O7. *Phys Rev B* **60**, R6980-R6983, (1999).

4 Snow, C. S. et al. Pressure-tuned collapse of the Mott-like state in Can+1RunO3n+1 (n=1,2): Raman spectroscopic studies. *Phys Rev Lett* **89**, (2002).

5 Karpus, J. F., Gupta, R., Barath, H., Cooper, S. L. & Cao, G. Field-induced orbital and magnetic phases in Ca3Ru2O7. *Phys Rev Lett* **93**, (2004).

6 Karpus, J. F. et al. Spectroscopic study of the field- and pressure-induced phases of the bilayered ruthenate Ca3Ru2O7. *Phys Rev B* **73**, (2006).

7 Peng, J. et al. From quasi-two-dimensional metal with ferromagnetic bilayers to Mott insulator with G-type antiferromagnetic order in $Ca_3(Ru_{1-x}Ti_x)_2O_7$. *Phys Rev B* **87** (2013).

8 Zhou, M. J. *et al.* First-principles lattice dynamics and thermodynamic properties of pre-perovskite PbTiO3. *Acta Mater* **171**, 146-153 (2019).

9 He, L. H. *et al.* Accuracy of generalized gradient approximation functionals for density-functional perturbation theory calculations. *Phys Rev B* **89** (2014).

10 Savitzky, A. & Golay, M. Smoothing and Differentiation of Data by Simplified Least Squares Procedures. *Analytical Chemistry* **36**, 1627-1639 (1964).

11 Devereaux, T. P. & Hackl, R. Inelastic light scattering from correlated electrons. *Rev Mod Phys* **79**, 175-233 (2007).

12 Valenzuela, B., Calderon, M. J., Leon, G. & Bascones, E. Optical conductivity and Raman scattering of iron superconductors. *Phys Rev B* **87** (2013)

13 Georges, A., de' Medici, L. & Mravlje, J. Strong Correlations from Hund's Coupling. *Annu Rev Conden Ma P* **4**, 137-178, (2013).

14 Petkov, V. et al. Lattice distortions and the metal-insulator transition in pure and Ti-substituted Ca3Ru2O7. *J Phys-Condens Mat* 51, (2023).

15 Puggioni, D., Horio, M., Chang, J. & Rondinelli, J. M. Cooperative interactions govern the fermiology of the polar metal Ca3Ru2O7. *Phys Rev Res* **2** (2020).

16 Yuan, Y. K. *et al.* Ultrafast quasiparticle dynamics in the correlated semimetal Ca3Ru2O7. *Phys Rev B* **99**, (2019).
